# Comparative In Vitro Antioxidant Capacity and Terpenoid Profiling of Pumpkin Fruit Pulps from a Serbian *Cucurbita maxima* and *Cucurbita moschata* Breeding Collection

**DOI:** 10.3390/antiox10101580

**Published:** 2021-10-07

**Authors:** Milorad Miljić, Gabriele Rocchetti, Sanja Krstić, Aleksandra Mišan, Milka Brdar-Jokanović, Fabio Marcheggiani, Erika Martinelli, Luigi Lucini, Elisabetta Damiani

**Affiliations:** 1 Department of Chemistry, Biochemistry and Environmental Protection, Faculty of Sciences, University of Novi Sad, 21102 Novi Sad, Serbia; milorad.miljic@dh.uns.ac.rs; 2 Department for Sustainable Food Process, Università Cattolica del Sacro Cuore, 29122 Piacenza, Italy; gabriele.rocchetti@unicatt.it (G.R.); erika.martinelli1@unicatt.it (E.M.); luigi.lucini@unicatt.it (L.L.); 3 FINS—Institute for Food Technology, 21000 Novi Sad, Serbia; aleksandra.misan@fins.uns.ac.rs; 4 Institute of Field and Vegetable Crops, National Institute of the Republic of Serbia, 21101 Novi Sad, Serbia; milka.brdar@ifvcns.ns.ac.rs; 5 Department of Life and Environmental Sciences, Polytechnic University of the Marche, 60131 Ancona, Italy; f.marcheggiani@univpm.it

**Keywords:** pumpkins, antioxidants, lipidomics, terpenoids, multivariate statistics

## Abstract

Pumpkin is considered a healthy and functional food. The consumption of pumpkins and pumpkin-based foods has been shown to confer several beneficial effects on human health due to their antioxidant capacity and terpenoid content. Consequently, this study aimed to characterize the in vitro antioxidant capacity (using FRAP and ABTS assays), terpenoid profile (using an untargeted lipidomics approach via high-resolution UHPLC-Orbitrap mass spectrometry), and carotenoid content (by HPLC-DAD) in pumpkin fruit pulp from accessions differing for species (11 *Cucurbita maxima* and 9 *Cucurbita moschata*), cultivar, and origin, belonging to a Serbian breeding collection. These accessions are candidates for inclusion within programs intended to improve pumpkin fruit quality. The results obtained in this work allowed us to highlight the best marker compounds, discriminating both the region of accession collection or breeding (“origin”) and the plant species. Furthermore, our findings have helped to identify the most suitable antioxidant-rich varieties to select for national breeding programs for improving human health. These findings provide valuable information to the overall current understanding of the potential health benefits of pumpkins and the discriminant triterpenoids underlying the *C. maxima* and *C. moschata* accessions investigated here, which include those of Serbian and non-Serbian origin.

## 1. Introduction

Pumpkins are annual vines or trailing plants that originated in Central to South America but are grown and consumed worldwide [1]. They belong to the genus *Cucurbita* of the *Cucurbitaceae* family, similar to melons, cucumbers, zucchini, etc. [2]. Interestingly, all parts of the plant from the Cucurbitaceae family are edible (seeds, flowers, roots, leaves, and fruits), and some parts such as flowers (pumpkins) and root (chayotte) can be found as ingredients in traditional cuisine [3]. 

Pumpkins are considered as healthy and functional food, and the consumption of pumpkins and pumpkin-based foods has been shown to confer several effects on human health, including hepatoprotective effects, antihyperglycaemic (antidiabetic) activity, anti-ulcer activity, anti-inflammatory activity, effects on prostatic hyperplasia (BPH) and urinary function, anti-microbial activity, and anticancer/antitumour effects [4,5,6,7,8,9]. The main factors that contribute to the nutritional and medicinal value of pumpkin fruits are their high total content of carotenoids and the presence of pectin and non-pectin polysaccharides, vitamins (A, C, E), dietary fibres, minerals (K, P, Mg, Fe, and Se), phenolic compounds (flavonoids, phenolic acids), and other compounds that possess health benefits [10,11,12,13,14,15]. However, the pumpkin fruit is also categorized as a functional antioxidant food due to various bioactive compounds such as polyphenols, triterpenoids, flavonoids, coumarins, cucurbitacins, and carotenoids, having significant antioxidant activity [16]. Indeed, by neutralizing free radicals and reactive oxygen species (ROS), antioxidants protect against oxidative damage to cells and tissues, an underlying cause of various chronic diseases such as cancer, cardiovascular diseases, diabetics, chronic inflammation, and other degenerative human diseases [17]. While most studies on the antioxidant activity of pumpkins have focused on seeds and leaves [18,19,20,21], there is almost no data on the antioxidant activity of fruits and their products [3,22]. Of the three most popular species of *Cucurbita* (*C. moschata*, *C. maxima*, and *C. pepo*) [10], *C. moschata* and *C. maxima* fruits possess good antioxidant activity, which is significantly correlated with their increased content in neoxanthin, violaxanthin, lutein, *β*-carotene, galactose, glucose, and dry matter content [22]. The total carotenoid content in *C. maxima* is usually higher than that found in *C. moschata* and *C. pepo*. In *C. maxima*, *β*-carotene, lutein, and violaxanthin are the major carotenoids; in *C. pepo*, the two dominant detected carotenoids are lutein and *β*-carotene, whereas in *C. moschata*, α-carotene and *β*-carotene are the major carotenoids [23]. However, it is worth bearing in mind that the antioxidant capacity of pumpkin fruits reported (mainly evaluated by using in vitro spectrophotometric assays) varies according to the antioxidant assay used. In this regard, in a previous work, *C. maxima* showed high values using the FRAP and CUPRAC antioxidant assays, whereas extremely lower antioxidant capacity values were measured by the DPPH assay [24].

Based on the aforementioned information on pumpkins as related to their nutritional importance in the human diet, this study aimed to characterize the in vitro antioxidant capacity, as well as the terpenoid profile (using an untargeted lipidomics approach via high-resolution UHPLC-Orbitrap mass spectrometry) of fruit pulp obtained from 11 *C. maxima* and 9 *C. moschata* accessions selected as potential candidates for breeding programs in Serbia intended to improve fruit quality. In this regard, while there are several reports on the content of terpenoids (mainly carotenoids) and polyphenols in pumpkins [25,26,27,28], very few studies have investigated their untargeted lipidomic profile and hence triterpenoid content using UHPLC-Orbitrap mass spectrometry. To the best of our knowledge, there are no investigations in the scientific literature on pumpkins and butternut squash from Serbian collections, which would help to identify the most suitable cultivars for breeding programs. On this basis, the final aim of our work was to highlight antioxidant-rich accessions potentially able to improve human health because of their rich and various terpenoid profiles. Finally, potential marker compounds (among the identified terpenoids) of both the region of accession collection or breeding (“origin”) and “the plant species” have been investigated by using multivariate statistical elaborations. The final aim of this work was to add value to the overall current understanding of the potential health benefits of pumpkins.

## 2. Materials and Methods

### 2.1. Chemicals and Equipment

Chemicals and solvents were purchased from Merck Life Science S.r.l. (Milan, Italy) and were of the highest analytical grade, including 2,4,6-tris(2-pyridyl) s-triazine (TPTZ), L-ascorbic acid, ferric chloride, 2,2’-azinobis-(3-ethylbenzothiazoline-6-sulfonic acid) diammonium salt (ABTS), 6-hydroxy-2,5,7,8-tetramethylchroman-2-carboxylic acid (Trolox), potassium persulfate (K_2_S_2_O_8_), and sodium carbonate. For HPLC analysis, *β*-carotene standard (>98% purity) was purchased from Sigma-Aldrich (Darmstadt, Germany). Ultrapure water was generated from a Milli-Q system by Merck Millipore (Merck KGaA, Darmstadt, Germany) and was used for all the experiments. Spectrophotometric measurements were recorded on a microplate reader (Synergy HT, Biotek, Winooski, VT, USA). For the lipidomics analysis, the following reagents (all LC–MS grade, purchased from Merck (Merck KGaA, Darmstadt, Germany) were used: isopropanol, methanol, and water. Finally, the phase modifiers, namely formic acid and ammonium formate, were purchased from Merck (Merck KGaA, Darmstadt, Germany).

### 2.2. Pumpkin Samples and Preparation

**Figure 1:**
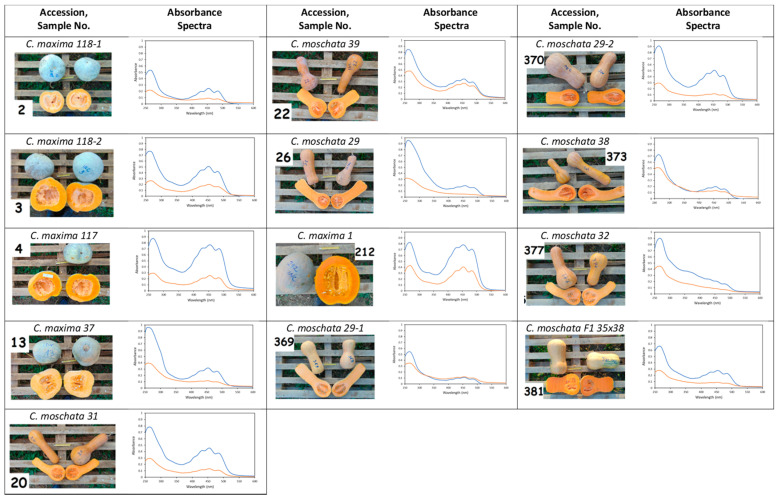
Pumpkins collected or bred in Serbia: images, sample number (in bold) associated with plot number in which each variety was grown, and absorbance spectra (blue line = 1st extraction; orange line = 2nd extraction. See Section 2.3). The absorbance spectra cover the absorbance value ranging from 0 to 1 (increments of 0.1) and from 250 to 600 nm (increments of 50 nm). Pumpkin calibres are more clearly visualized in Figure S1 (Supplementary material) and weight, length, and diameter are reported in Table S3 (Supplementary material).

**Figure 2:**
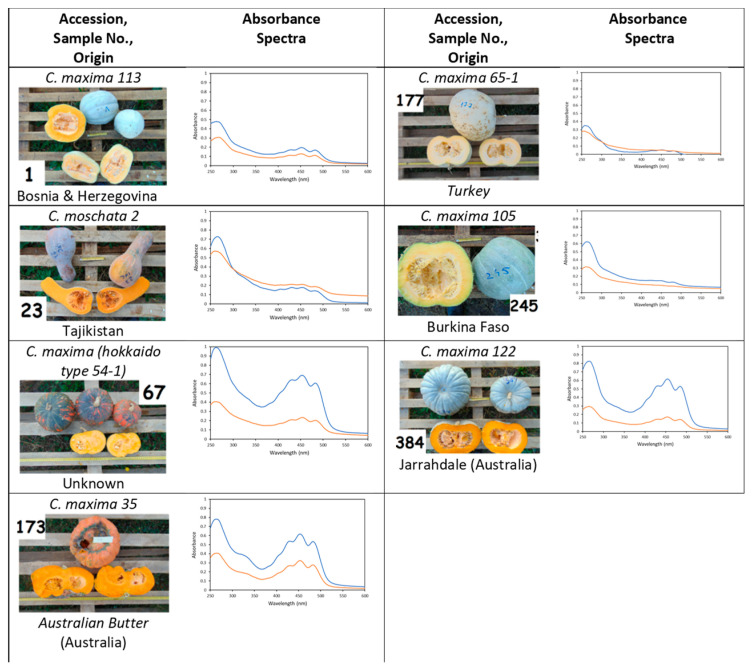
Pumpkins not originally from Serbia: images, sample number (in bold) associated with plot number in which each variety was grown, geographical origin (when known), and absorbance spectra (blue line = 1st extraction; orange line = 2nd extraction. See Section 2.3). The absorbance spectra cover the absorbance values ranging from 0 to 1 (increments of 0.1) and from 250 to 600 nm (increments of 50 nm). Pumpkin calibres are more clearly visualized in Figure S2 (Supplementary material), and weight, length, and diameter are reported in Table S3 (Supplementary material).

Twenty studied accessions, reported in Figure 1 and Figure 2, were grown and harvested in Bački Petrovac, North Serbia. Pumpkins and squashes were grown under standard production conditions for Serbia, except for the distances between the rows, which were 5 m. Mineral fertilizer was added to the soil, there was no irrigation since there was enough moisture in the soil, and pesticides were not applied because there were no significant diseases. To prevent and protect against weeds, inter-row cultivation was performed on two occasions and within the rows, and weeds were destroyed manually. Sowing was performed in early May, and fruits were harvested from the end of September to late October 2018. Pumpkin pulp samples were taken from 5–8 fruits within the same plot (plot number = sample number). The fresh pumpkin samples (50 g each) were then freeze-dried at –80 °C on a Christ Alpha 1-2 LD Freeze Dryer (Switzerland) for 48 h.

The material was from 20 accessions from the breeding collection of the Institute of Field and Vegetable Crops, National Institute of the Republic of Serbia, Novi Sad (Serbia). The accessions were chosen based on previous research to include in the study broader variability regarding pumpkin pulp carotenoid content. All the accessions were characterized by at least one of the desirable agronomic traits, such as fruit yield, fruit shelf life, earliness, disease, heat, or drought tolerance. Eleven accessions were of *Cucurbita maxima* Duchesne species (5 populations from Serbia, 1 population from Bosnia and Herzegovina, 1 population from Turkey, 1 population from Burkina Faso, 1 line from the Institute's breeding program devoted to developing a quality cultivar of hokkaido type, and 2 cultivars, namely “Australian butter” and “Jarrahdale”), whereas 9 accessions were of *Cucurbita moschata* Duchesne species (6 populations collected in Serbia, 1 population from Tajikistan, and 1 line and 1 hybrid from the Institute's breeding program devoted to developing quality cultivars of butternut type). All the *C. maxima* populations are very popular in Serbia; fruits are medium-sized, usually transverse broad elliptic, circular, or broad elliptic shaped, with lighter grey skin, like Jarrahdale, however, without or with very shallow grooves. All the *C. moschata* accessions are of the butternut type. The populations from Serbia and Bosnia and Herzegovina were collected within the Institute's regular activities. The populations from Turkey, Burkina Faso, and Tajikistan were obtained via the Germplasm Resources Information Network (GRIN), courtesy of the Agricultural Research Service, United States Department of Agriculture (*codes PI 175703 07GI SD, PI 490350 07GI SD, and Grif 17270, respectively).

### 2.3. Pumpkin Pulp Extracts and Absorbance Spectra

Each sample (25 mg) was dissolved in 1 mL DMSO and incubated for 2 h at 50 °C with occasional vortexing. Samples were then centrifuged at 1350× *g* for 5 min at room temperature, the supernatants were collected, and their absorbance spectra were scanned from 250 to 600 nm in quartz cuvettes on a Shimadzu UV-2401PC spectrophotometer (against a blank containing DMSO). The same procedure reported above was repeated on the remaining pellet for a second round of extraction.

### 2.4. Determination of Antioxidant Capacity (ABTS, FRAP)

To evaluate the in vitro antioxidant capacity of the pumpkin extracts, two different methods were employed, namely ABTS and FRAP (ferric ion reducing antioxidant power) assays, as essentially described in [29,30]. For these assays, the DMSO solutions obtained after the first and second pumpkin extractions reported above were mixed in a 1:1 ratio, and 400 µL of this solution was diluted with 500 µL water.

To generate the ABTS^+^^•^ coloured radical cation stock solution, a 7 mM aqueous ABTS solution and a 24.5 mM aqueous K_2_S_2_O_8_ solution were first prepared. These were mixed in a 9:1 ratio, respectively, and left to stand in the dark overnight at room temperature (12–16 h). The prepared ABTS^+•^ stock solution was then diluted 50–60× with water, and the absorbance was checked at 734 nm, as a value of 0.9 ± 0.1 should have been reached for the working solution. For the standard, an ethanolic stock solution of Trolox (1.8 mM) was appropriately diluted in water to obtain 7 increasing concentrations ranging between 0 and 0.3 mM. The blank sample contained DMSO/water 4:5 ratio. Then 30 µL of pumpkin extract/blank/standard were each transferred to a transparent 96-well microplate to which 270 µL of working ABTS^+•^ solution was added. The microplate was shaken and left in the dark for 2 h at room temperature prior to reading the absorbance at 734 nm against water. The results are expressed as mM Trolox equivalents (TXE), using the linear regression deriving from the standard curve.

For the FRAP assay, the following solutions were prepared: 10 mM TPTZ dissolved in 40 mM HCl, 20 mM FeCl_3_ in water, 300 mM acetate buffer pH 3.6, standard aqueous stock solution of ascorbic acid (1.13 mM) appropriately diluted in water to obtain 8 increasing concentrations ranging between 0 and 0.2 mM, and a blank sample containing DMSO/water in a 4:5 ratio. The FRAP working reagent was then prepared by mixing TPTZ/FeCl_3_/acetate buffer in the ratio of 5:5:50 immediately before measurement. This solution was added to each well of a 96-microplate already containing 10 μL of pumpkin extract/blank/standard. The microplate was shaken, and absorbances were read at 593 nm after 6 min of incubation. The results are expressed as mM ascorbic acid equivalents (AAE), using the linear regression derived from the standard curve.

### 2.5. Lipidomic Profiling by UHPLC-Orbitrap Mass Spectrometry

To extract the lipophilic compounds, a 200 mg of lyophilized sample was weighed and dissolved in 5 mL of a solvent mixture consisting of tert-butyl methyl ether (MTB) and 80% aqueous methanol (1:1, *v*/*v*). The samples were then mixed by vortexing for 3 min and then extracted using ultrasound-assisted extraction for 10 min. Following a centrifugation step (10 min at 4 °C, 7000× *g*), 300 µL of the supernatant were taken and evaporated until dryness. Samples were then resuspended in 300 µL of a solution consisting of 65% isopropanol, 30% methanol, and 5% water and transferred to a 2 mL vial. The UHPLC-HRMS analyses were done immediately after the extraction process.

Terpenoids were profiled through a UHPLC-MS lipidomics-based approach, based on a Q Exactive™ Focus Hybrid Quadrupole-Orbitrap Mass Spectrometer (Thermo Scientific, Waltham, MA, USA) coupled to a Vanquish ultra-high-pressure liquid chromatography (UHPLC) pump and equipped with a HESI-II probe (Thermo Scientific, Waltham, MA, USA [31]). In this regard, a BEH C18 (2.1 × 100 mm, 1.7 µm) analytical column maintained at 40 °C was used. The mobile phases consisted of (A) water/methanol (95/5, *v*/*v*) and (B) 2-propanol/methanol/water (65/30/5, *v/v/v*). Both phases were modified with 5 mM ammonium formate and 0.1% formic acid. The detailed parameters related to both linear gradient and flow rate can be found elsewhere [31].

The full scan MS analysis considered both positive and negative ionization with a typical mass resolution of 70,000 at *m*/*z* 200. In our experimental conditions, pooled quality control (QC) samples were randomly injected through the sequence and analysed in a data-dependent (Top N = 3) MS/MS mode. For this latter, the mass resolution was reduced to 17,500 at *m*/*z* 200. The parameters related to automatic gain control targeted (AGC) and maximum injection time for both MS and MS/MS modes have been previously optimized [31]. Regarding data-dependent MS/MS, the Top N ions were fragmented according to stepped normalized collision energies, namely 10, 20, and 40 eV. The injection volume was 6 μL considering a full-scan acquisition of 150–1500 *m*/*z*, with a randomized injection sequence. The heated electrospray ionization (HESI) parameters were optimized in previous work [32]. In addition, the instrument was calibrated using Pierce™ positive and negative ion calibration solutions (Thermo Fisher Scientific, San Jose CA, USA).

The post-acquisition workflow was based on two open source software, namely MS-DIAL (version 4.38) and MS-Finder [33,34]. In this regard, the annotation step was done according to spectral matching against the comprehensive database LipidBlast, excluding the retention time information from calculating the total identification score. Therefore, the putative annotation step was based on mass accuracy, isotopic pattern, and spectral matching in our experimental conditions. Finally, the software MS-Finder was used for in silico fragmentation of the not fully annotated MS/MS features, according to the structures reported on Lipid Maps and FoodDB libraries (available in the same software).

### 2.6. Carotenoid Analysis and Quantification by HPLC-DAD

Peeled and chopped pumpkin fruit (3 g) was homogenized in 10 mL of solvent (n-hexane:dichloromethane; 1:1, *v*/*v*), using an Ultra Turrax® IKA® T18 basic. It was then centrifuged at 7000× *g* for 15 min at 5 °C. The liquid phase was separated, and the procedure was repeated 2 more times. After that, 20 mL of solution was collected and evaporated using a dryer (UF55 universal oven, Memmert GmbH + Co. KG). The dry residue was dissolved in 1 mL of methanol and analysed by HPLC-DAD. Carotenoids were separated, identified, and quantified following the method of Morais et al. [35] and Kevrešan et al. [36] on an Agilent 1200 series HPLC system with DAD detector equipped with an Agilent, Eclipse Plus C18 (5.0 µm; 3.0 × 250 mm) column. Two eluents were used, namely (A) acetone/water (75:25, *v*/*v*) and (B) acetone/methanol (75:25, *v*/*v*), with the following gradient: from 0 to 25% B in 10 min, from 25 to 100% B in 35 min, 100% B for 10 min, and a flow rate of 1.5 mL/min at 24 ± 1 °C. Carotenoids were detected at 460 ± 4 nm. For each peak, the whole spectrum (from 350 to 600 nm) was recorded. Peaks were identified by comparing their retention time and spectra with literature data and calculated as *β*-carotene equivalents.

### 2.7. Statistical Analysis

In this work, the ABTS and FRAP assays were performed in triplicate as three independent experiments where each sample was included in duplicate, and the results are reported as means ± standard deviation (SD). The results were statistically analysed using EXCEL with installed DSAASTAT add-in. To determine statistically significant differences between varieties, an analysis of variance was made. Multiple comparisons analyses were performed using the Tukey HSD method (*p* < 0.05). Pearson’s correlations were calculated using the Excel CORREL function.

Regarding the statistical elaboration of the HRMS data, a supervised orthogonal partial least squares discriminant analysis (OPLS-DA) was carried out using SIMCA 13 software (Umetrics, Malmo, Sweden). The OPLS-DA model was cross validated and inspected for outliers. After that, model parameters related to goodness of fit and goodness of prediction (R^2^Y and Q^2^Y, respectively) were recorded. The variables importance in the projection (VIP) approach were finally used to select those terpenoid compounds possessing the highest discrimination potential (VIP score > 0.8) in the predictive models built considering both plant species and “the origin” as class discrimination criteria. Finally, a Venn diagram was inspected to evaluate those compounds varying exclusively as a function of the two parameters under investigation.

## 3. Results

### 3.1. Absorbance Spectra of Pumpkin Pulp Extracts

The absorbance spectra of the pumpkin pulp extracts reported in Figure 1 and Figure 2 were characteristic of carotenoids that reflected the organization of their conjugated carbon–carbon double bond system. Indeed, the absorption spectra of carotenoids usually have three maximum bands between 400 and 550 nm, of which the central ones are strongest. In certain cases, the first and/or third peaks were difficult to observe, as in samples 245 and 377. Furthermore, the number of conjugated double bonds determines the wavelength of maximum peak absorption, with higher numbers leading to a longer shift in wavelength maxima and a redder appearance of the carotenoid [37]. From comparing the absorbance spectra for the different pumpkin pulps, one can observe that they all had a maximum peak in the visible region centred around 450 nm, which was attributed to carotenoids containing between 9 and 10 conjugated double bonds [37]. However, the degree of absorbance differed greatly among most samples, ranging from ~0.8 (sample 212) to 0.1 (sample 177) for the first extraction. A second round of extraction was also performed to ensure maximum extraction of carotenoids, and these two extracts were combined in a 1:1 ratio for analysis of antioxidant capacity. The spectra in Figure 1 and Figure 2 also presented an additional band in the UV region characteristic of the *cis*-carotenoid isomers. One might expect that the colour intensity of the pumpkin pulp should correlate with the absorption spectra maximum, and although in most cases this was observed, a few exceptions could be noted. For example, sample 67 had extremely pale pulp, but it displayed a high absorbance between 400 and 500 nm, whereas sample 23, which had bright orange pulp, has a low absorbance in this range. Hence, the colour of the pumpkin pulp does not necessarily reflect the carotenoid content present, as was confirmed from HPLC-DAD data (discussed later).

### 3.2. In Vitro Antioxidant Capacity of Pumpkin Pulp Extracts

**Figure 3:**
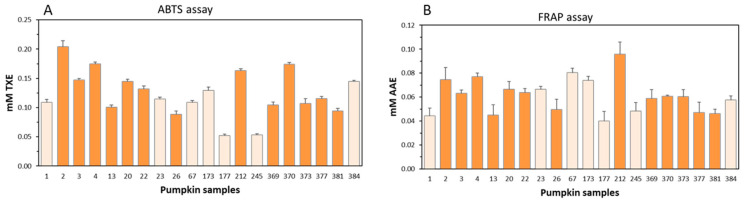
Antioxidant capacity of pumpkin samples. Antioxidant capacity was measured using the ABTS (**A**) and FRAP (**B**) assays. The dark-orange-coloured bars indicate pumpkin samples of Serbian origin, while light-orange-coloured bars indicate pumpkins not originally from Serbia. Error bars represent ± SD of the mean value, *n* = 3 independent experiments in which each sample was analysed in duplicate. TXE = Trolox equivalents; AAE = ascorbic acid equivalents.

To obtain a comprehensive picture of the in vitro antioxidant capacity of the pumpkin pulp extracts, two different spectrophotometric methods were employed, which differ in their determination principles. The ABTS assay is based on both SET (single electron transfer) and HAT (hydrogen atom transfer) mechanisms, where the ABTS^+•^ radical may be neutralized either by direct reduction via electron transfer or by radical quenching via H atom transfer. The FRAP assay is instead mainly based on the SET mechanism [38]. The results are reported in Figure 3, and statistically significant differences among the accessions are reported in Table S1 (Supplementary material). Figure 3 shows large variability in antioxidant capacity among the different samples using both assays, ranging between 0.20 and 0.05 mM TXE (ABTS assay) and between 0.10 and 0.04 mM AAE (FRAP assay). The results obtained showed that the antioxidant capacity of pulps of Serbian pumpkins was comparable to those originating from other countries. Indeed, with the ABTS assay, the top four samples displaying the highest antioxidant capacity, namely 2, 4, 212, and 370, were significantly different from those of non-Serbian origin, whereas those displaying the statistically lowest antioxidant capacity were both of non-Serbian origin (samples 177 and 245). Indeed, sample 177 originating from Turkey showed the lowest antioxidant potential (ABTS: 0.052 mM TXE; FRAP: 0.04 mM AAE). With the FRAP assay, these differences were not as remarkable. Although sample 212 of Serbian origin displayed the highest antioxidant activity (0.10 ± 0.01), it was not statistically different from the non-Serbian sample, 67 (0.08 ± 0.004). The relatively similar results with the two assays were also reflected in the good correlation value obtained (ABTS:FRAP = 0.672).

To gain insights into the in vitro antioxidant capacity of the pumpkin pulp samples, lipidomic profiling (focused on the terpenoids) and carotenoid content were assessed using Orbitrap HRMS and HPLC-DAD, respectively.

### 3.3. Untargeted Lipidomic Profiling and Discrimination of Pumpkin Pulp Extracts

**Figure 4:**
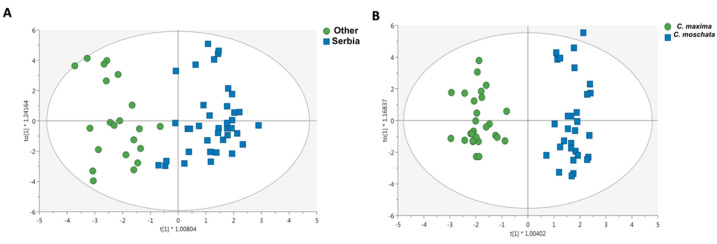
OPLS-DA score scatter plot obtained considering the discrimination based on the lipidomic profiles of the pumpkin samples originally from Serbia and those originating outside Serbia (**A**), and when considering the plant species (**B**), i.e., *C. maxima* vs. *C. moschata*.

The untargeted lipidomic profiling based on UHPLC-Orbitrap MS allowed us to putatively identify 44 dietary terpenoids, which are reported in Table S2 (Supplementary material), together with their annotation parameters (such as MS and MS/MS spectra). Overall, among the annotated compounds, we found 4 carotenoids, 2 cucurbitacins, 11 diterpenoids, 4 monoterpenoids, 1 sequaterpenoid, 11 sesquiterpenoids, 2 sesterterpenoids, 8 triterpenoids, and 1 xanthophyll. To better discriminate the potential impact of geographical origin and plant species on the terpenoid profile detected, a supervised OPLS-DA multivariate statistical approach was used. The OPLS-DA score plot represented in Figure 4A shows that relatively clear differences exist between the pumpkins originally from Serbia and those from other countries, independently of plant species. On the other hand, Figure 4B also shows that the differences amongst the two plant species under investigation are visible and imposed by the terpenoid profile. Therefore, to evaluate the importance in projection of the two different OPLS-DA models built, the VIP selection method was used, and the discriminant terpenoids (VIP score > 0.8) are reported in Table 1 and Table 2, when considering the “origin” and the “plant species”, respectively.

Regarding the first OPLS-DA model considered (as a function of the origin) and reported in Figure 4A, we found more than acceptable goodness parameters, namely the R^2^Y = 0.807 (goodness of fit) and the Q*^2^*(cum) = 0.589 (goodness of prediction). This prediction model was characterized by 24 discriminant compounds (as reported in Table 1), with a clear abundance of sesquiterpenoids (7 compounds), followed by 5 diterpenoids, and other compounds. The highest discrimination potential was assigned to myrigalone A (VIP score = 1.77), belonging to monoterpenoids. In addition, two carotenoids (namely β-carotene and violaxanthin) showed a VIP score > 1, thus being highly affected by the origin. Finally, among the cucurbitacins (typical compounds in pumpkins), we found a strong discrimination degree for cucurbitacin E (VIP score = 1.57). Regarding the second OPLS-DA model built (i.e., discriminating *C. moschata* vs. *C. maxima*; Figure 4B), we found a higher prediction ability, namely the Q^2^(cum) = 0.853, with a goodness of fit (R^2^Y) = 0.937. As reported in Table 2, the VIP selection method identified 26 terpenoids as the most discriminant. In this regard, sesquiterpenoids were again the most numerous discriminant compounds (8), followed by diterpenoids (7 compounds). Accordingly, the highest VIP score was recorded for epioxylubimin (2.22) belonging to the sesquiterpenoids subclass. Interestingly, cucurbitacin E was highlighted as a specific marker of the species *C. maxima*, characterized by a VIP score = 1.09 and a LogFC = 2.06.

**Figure 5:**
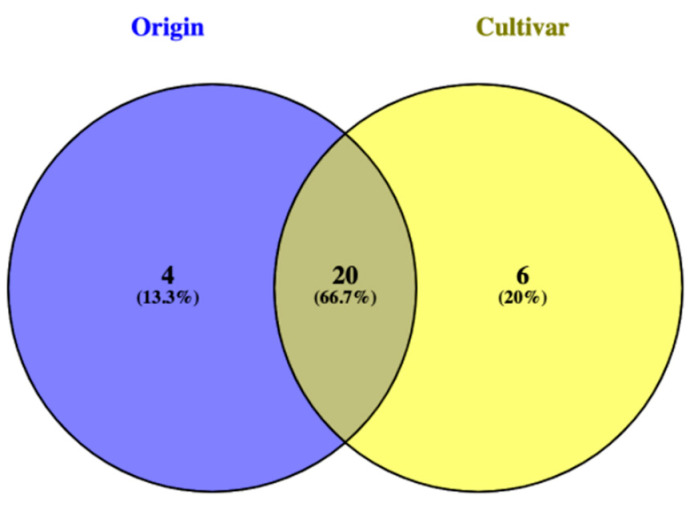
Venn diagram considering the VIP discriminant compounds according to either “origin” or “plant species”.

Finally, a Venn diagram was used to discern among the VIP marker compounds exclusively representing the two conditions under investigation. As shown in Figure 5, 66.7% of the VIP compounds were shared, with some markers exclusively characterizing origin (4 VIP) and plant species (6 VIP). The exclusive markers of “origin” were (9E)-valenciaxanthin, 19'-hexanoyloxymytiloxanthin, violaxanthin, and cincassiol B, while those characterizing the “plant species” discrimination were 8-alpha-8-hydroxy-12-oxo-13-abieten-18-oic acid, apo-10'-violaxanthal, apo-12'-violaxanthal, glandulone B, methyl (9Z)-6'-oxo-6,5'-diapo-6-carotenoate, and ganoderiol C.

### 3.4. Carotenoid Contents in Pumpkin Pulp Extracts

**Figure 6:**
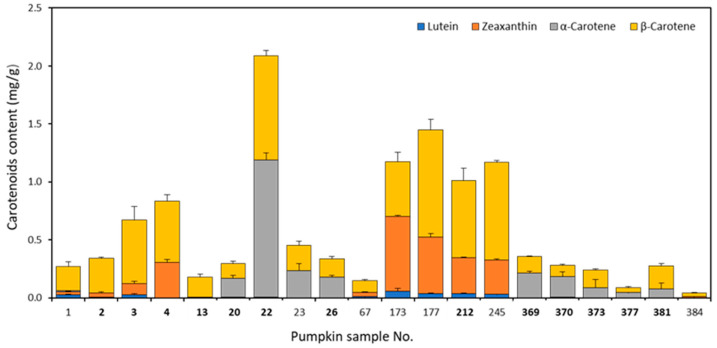
Total carotenoid content in pumpkin samples measured by HPLC-DAD. Results are expressed as mg/g dry matter. The cumulative carotenoid content for each sample is broken down into the four carotenoids calculated (lutein, zeaxanthin, α-carotene, β-carotene). Serbian samples are indicated in bold. Results are expressed as mean values ± S.D (*n* = 2).

The carotenoid contents in the pumpkin samples were analysed by HPLC-DAD, and the results are reported in Figure 6. The data show that there was a large variability in the cumulative total carotenoids content (CC), ranging from 2.09 (sample 22) to 0.04 (sample 384) mg/g dry matter, with more than half the samples bearing a total cumulative content <0.5 mg/g. The distinct values of CC for each access were likely due to genetic influence rather than climate, soil, sowing, harvesting, and storage conditions, since these variables were the same for all the samples. The five accessions with a CC above 1.0 mg/mL (22, 173, 177, 212, 245) had similar contents of β-carotene, except for sample 173, which had slightly less. In general, one might expect that the pulp of these pumpkins would be highly coloured, since β-carotene is mainly responsible for the bright orange–red colour of pumpkin pulps [39]. Instead, the pulp of accessions 177 and 245 was very pale (Figure 2). From this, one can infer that β-carotene content and visual colour are not directly related, at least not amongst the accessions investigated in this study. Correlations between CC and antioxidant activity were also carried out, and from the results obtained (ABTS:CC = −0.155, FRAP:CC = 0.097) do not appear to be correlated.

The graph also shows large variability in the four types of carotenoids quantified, with sample 22 having the highest content of α-carotene and among the highest for β-carotene, but zeaxanthin and lutein were hardly detected. The most consistent carotenoid detected was β-carotene, found in all samples, albeit at different concentrations. Interestingly, zeaxanthin was detected in all pumpkins not originally from Serbia, except in two (23 and 384), whereas in Serbian pumpkins, this carotenoid was present in only four samples (2, 3, 4, 212) out of the 13 tested.

## 4. Discussion

Previous studies have reported that pumpkins and their by-products show various beneficial impacts on human health, as they are characterized by a high content of bioactive compounds such as phenolic compounds, carotenoids, and others, which can protect human cells from the action of free radical (oxygen and non-oxygen) species [27,40]. The biopotential of pumpkin seeds and vegetative parts has been well-studied and is receiving growing attention as a good candidate for functional food formulations [21]. However, there is a lack of research on antioxidant activity and other biological activities of pumpkins fruit pulp. Hence, the present investigation on in vitro antioxidant activity of pumpkin accessions from a Serbian breeding collection has allowed us to identify those with the greatest in vitro antioxidant potential and carotenoid content. In accordance with statistical analysis and multiple comparison approach, the overall results obtained, using ABTS and FRAP antioxidant assays, indicate that the pumpkin accessions **212, 4,** and **2**, all collected or bred in Serbia, stand out as having amongst the highest antioxidant capacity. This is an important finding as it gives indications of the biopotential of pumpkins through selective plant breeding that could have implications for improving human nutrition. Additionally, when comparing the antioxidant capacity of the pulp extracts of the different pumpkin species, the *Cucurbita maxima* extracts showed higher values than *Cucurbita moschata*. This is in agreement with previous studies, where a similar outcome was observed between the two species [22,41]. Indeed, several studies indicated that *C. maxima* is the most investigated pumpkin species, likely due to its greater bioactive potential [27,32]. However, since different experimental conditions, together with extraction methods and antioxidant assays, were applied amongst the studies, direct comparison of the values reported in the literature with those presented in this study is not possible.

The antioxidant capacity is strictly dependent on the content of bioactive compounds. The literature data indicates that carotenoids are one of the dominant bioactive compounds in pumpkins [41,42,43,44], and accordingly they were quantified in the present study. According to the Pearson's correlation coefficients between the total carotenoids and antioxidant activity values, we detected negative or negligible not significant values for both ABTS (−0.155) and FRAP (0.098). Although pumpkins are considered an excellent source of carotenoids, the results obtained in this study apparently seem to indicate that carotenoids do not play a major role in the antioxidant capacity in the accessions selected. It is well-known that carotenoids are generally very unstable molecules. Therefore, storage, sample preparation, and other unidentified factors could account for these not significant correlation values [23,45]. However, it is possible that the different phytocompounds as discerned by the discriminant terpenoids identified by the VIP approach reported in Table 1 and Table 2, on both the origin of the pumpkin accessions and on the two pumpkin species, respectively, could contribute to the in vitro antioxidant capacity values reported. Notwithstanding the results obtained, carotenoids are regarded as powerful antioxidants and as such can contribute to the prevention of aging and the development of some diseases such as atherosclerosis and other cardiovascular diseases [42,44,46]. They can also protect the eye and macula from degradation, with zeaxanthin being the predominant carotenoid of the macula pigment and therefore important for eye health [47].

In our study, the results of carotenoid quantification on the pumpkin accessions show that they decreased in the following order of concentration: *β*-carotene > α-carotene > zeaxanthin > lutein. These four carotenoids were chosen since they are the most widespread among the Cucurbitaceae family [48]. The total carotenoids found in the present study are similar to those reported in the literature for pumpkin products from *C. maxima* and *C. moschata*, such as in pumpkin pulp flour [23,49]. Interestingly, on comparing the pumpkin samples based on their origin, we noticed that, in general, those of Serbian origin were characterized by the presence of both *β*-carotene and α-carotene, while in those of non-Serbian origin, the presence of *β*-carotene and zeaxanthin was dominant. The results demonstrate that there is high diversity in carotenoid type and concentration in the different Cucurbitaceae species and cultivars, and this is in accordance with several literature reports. For example, Kulczynski and Gramza-Michałowska [27] found that lutein was the most abundant carotenoid among 11 *C. maxima* cultivars, contrary to our results, where it was detected in the lowest amount, whereas β-carotene was the most abundant among the *C. maxima* cultivars in our study. In a previous study by Kulaitiene et al. [50] on cultivars of the *C. maxima* species, lutein and zeaxanthin were the most abundant carotenoids. Azevedo-Meleiro and Rodriguez-Amaya [10] also found different carotenoids, depending on the pumpkin species and cultivar, with *C. moschata* cultivars having higher contents of α-carotene as well as *β*-carotene and smaller amounts of lutein and neoxanthin. On cultivars of *C. moschata* species, both Norshazila et al. [51] and de Carvalho et al. [52] observed that *β*-carotene was the predominant carotenoid. However, a study by Murkovic et al. [28] showed that *β*-carotene was the most dominant carotenoid in most tested pumpkin varieties in both *C. maxima* and *C. moschata* fruits from Austria. The above discrepancies may depend on the environmental and growing conditions (climate, soil, sowing, harvesting, storage). However, when such factors are limited as in the present study, the genotype becomes an important determinant that could affect the content of bioactive compounds in the pumpkin flesh. Therefore, at the present knowledge, no generalizations on the type of carotenoid or its content for the different species and cultivars of pumpkins can be made. A multiyear and preferably multilocation study involving the same set of accessions would provide the information required for discrimination between environmental and genetic factors contributing to pumpkin fruit quality parameters.

To gain further insights on the phytochemical composition of the accessions investigated, untargeted lipidomic profiling on the triterpenoids using UHPLC-Orbitrap mass spectrometry was conducted. Indeed, this is one of the few reports that has looked at the best discriminant terpenoid compounds potentially correlated to “origin” and “species”, and the first of its kind on Serbian pumpkin and butternut squash varieties. However, it is important to highlight that terpenoids are not the only contributors to the discrimination observed, considering that several other compounds and secondary metabolites (including polyphenols) could be particularly useful for this purpose. Looking at recently published papers, some authors investigated the impact of both cultivar and farming system on the nutritional composition of butternut squash (i.e., *Cucurbita moschata* D.) by highlighting the changes in amino acids, minerals, total phenolics and carotenoids (as evaluated by spectrophotometric assays), and vitamins [53]. In another study, Luo et al. [54] evaluated the accumulation of carotenoids in fruit flesh during fruit development in two *Cucurbita maxima* inbred lines; however, no reference to the terpenoid profiling was done. Indeed, according to Kulczynski and Gramza-Michalowska [27], the content of carotenoids in pumpkin has been documented in many publications, but so far there has been no complex analysis of the profile of other bioactive compounds, such as other terpenoids. In this regard, as reviewed by Montesano et al. [48], in addition to the tetraterpenes (represented essentially by carotenoids), pumpkins are also characterized by triterpenoids (such as cucurbitacins), diterpenes, and sesquiterpenes. However, more studies on robust analytical platforms are needed to extend the available information on the terpenoid profile [48]. The interest in terpenoids is mainly due to their potential medicinal value, considering that some compounds can produce a certain physiological effect in the human body. Among these beneficial compounds, several studies have been carried out to explore the health-promoting properties of cucurbitacins. These compounds are classified into twelve categories, involving cucurbitacins A–T, and differing with respect to oxygen functionalities at various positions and by the degree of glycosylation. In our experimental conditions, we have detected several terpenoid classes, such as carotenoids, cucurbitacins, diterpenoids, monoterpenoids, sesquaterpenoids, sesquiterpenoids, sesterterpenoids, and triterpenoids (supplementary material). Interestingly, cucurbitacin E was detected in all the samples under investigation and was found to be a discriminant marker of the plant species, showing higher values in *C. maxima* (Table S3). According to the literature, many pharmacological and clinical investigations have demonstrated that cucurbitacin E possesses various pharmacological activities, such as antioxidant, antimicrobial, antiulcer, antitumor, anti-hepatitis, and anti-hyperglycaemic effects [48,55]. However, further studies are required considering the potential toxicity effects of cucurbitacin E and its glycoside [56].

## 5. Conclusions

Pumpkin is considered a nutritionally rich food plant, potentially providing functional traits. However, like all plant-based foods, the actual content of functional components can be affected by species, cultivar, agronomic, and pedo-climatic conditions. Our work confirmed that both pumpkin species provide relevant amounts of carotenoids and a significant antioxidant capacity. Nonetheless, significant differences could be outlined across the tested accessions, allowing identification of the most suitable cultivars to select for breeding programs based on functional traits. The species tested provided high antioxidant capacity and β-carotene content, and a potential source of pro-vitamin A whose deficiency remains a major health problem worldwide. Lipidomics followed by multivariate statistics pointed out that functional components are affected by both the cultivar and the origin, with distinctive markers of each condition. In general, violaxanthin-related markers were the most represented discriminant compounds. The terpenoid cucurbitacin E was a specific marker of the species *C. maxima*, whereas the non-Serbian varieties investigated here are distinguished by their high content in zeaxanthin. This latter represents a valuable functional component with potential use in the pharmaceutical industry and in supplements used in the treatment of eye diseases. Taken together, our findings provide valuable information to the overall current understanding of the potential health benefits of pumpkins, as well as the discriminant triterpenoids underlying the *C. maxima* and *C. moschata* accessions investigated here, which include those of Serbian and non-Serbian origin.

## Figures and Tables

**Table 1:** Discriminant terpenoids according to the comparison between pumpkins originating from Serbia and those originating outside Serbia. Compounds were identified by the VIP (variable importance in projection) approach following OPLS-DA discriminant analysis. The VIP scores (a measure of variable’s importance in the OPLS-DA model) and the corresponding cv SE (cross-validated standard errors) together with the Log2 fold-change values (FC > 1.1, *p* < 0.05) for the comparison “Serbian” vs. “Other” pumpkins are also provided. ns = not significant.

VIP Marker (OPLS-DA)	VIP Score (OPLS-DA)	Log2(FC) Serbian vs. Other
Myrigalone A	1.77 ± 0.41	−0.46
Steviol	1.70 ± 0.53	−1.64
Sterebin A	1.69 ± 0.36	0.81
Ginsenoside Rh4	1.64 ± 0.76	0.26
Cucurbitacin E	1.58 ± 0.80	ns
Oxysolavetivone	1.44 ± 0.79	−0.96
ent-15-Kaurene-17,19-dioic acid	1.44 ± 0.49	0.46
12'-Apo-b-carotene-3,12'-diol	1.36 ± 0.82	−0.69
Apo-14'-zeaxanthinal	1.32 ± 0.42	−0.26
Epioxylubimin	1.30 ± 0.44	1.44
(8'R)-Neochrome	1.25 ± 0.62	0.19
(9E)-Valenciaxanthin	1.19 ± 1.07	−0.22
Lubiminol	1.18 ± 0.77	1.01
beta-Carotene	1.14 ± 0.74	−0.77
19'-Hexanoyloxymytiloxanthin	1.11 ± 0.66	ns
4-Methoxycinnamoyloleanolic acid methyl ester	1.08 ± 0.44	−0.49
7(14)-Bisabolene-2,3,10,11-tetrol	1.04 ± 0.91	ns
Geranyl benzoate	1.04 ± 0.54	ns
Violaxanthin	1.01 ± 0.54	ns
Cincassiol B	1.01 ± 0.42	0.61
Momordicoside C	0.99 ± 1.12	ns
Methyl geranate	0.95 ± 1.73	−0.46
Furanofukinin	0.89 ± 0.50	ns
4,5-Dihydrovomifoliol	0.85 ± 0.69	−0.39

**Table 2:** Discriminant terpenoids according to the comparison between the two different plant species, *C. maxima* and *C. moschata*. Compounds were identified by the VIP (variable importance in projection) approach following OPLS-DA discriminant analysis. The VIP scores (a measure of variable’s importance in the OPLS-DA model) and the cvSEs are also shown. The VIP scores (measure of variable’s importance in the OPLS-DA model) and the corresponding cvSEs (cross-validated standard errors) together with the Log2 fold-change values (FC > 1.1, *p* < 0.05) for the comparison *C. maxima* vs. *C. moschata* are also provided. ns = not significant.

VIP Marker (OPLS-DA)	VIP Score (OPLS-DA)	Log2(FC) *C. maxima* vs. *C. moschata*
Epioxylubimin	2.23 ± 0.42	−2.57
8alpha-8-Hydroxy-12-oxo-13-abieten-18-oic acid	1.77 ± 0.48	−0.71
Lubiminol	1.73 ± 0.41	−1.93
4-Methoxycinnamoyloleanolic acid methyl ester	1.63 ± 0.62	1.32
Sterebin A	1.53 ± 0.33	−0.67
12'-Apo-b-carotene-3,12'-diol	1.53 ± 0.73	1.11
Ginsenoside Rh4	1.36 ± 0.36	ns
ent-15-Kaurene-17,19-dioic acid	1.35 ± 1.21	ns
Geranyl benzoate	1.18 ± 0.94	0.39
Apo-14'-zeaxanthinal	1.14 ± 0.60	1.19
(8'R)-Neochrome	1.13 ± 0.85	ns
Steviol	1.10 ± 1.06	0.69
Cucurbitacin E	1.09 ± 1.36	2.06
beta-Carotene	1.00 ± 1.27	0.93
Apo-10'-violaxanthal	0.96 ± 0.84	1.19
Momordicoside C	0.95 ± 0.77	ns
Myrigalone A	0.94 ± 0.71	0.47
7(14)-Bisabolene-2,3,10,11-tetrol	0.90 ± 0.69	−0.16
Apo-12'-violaxanthal	0.90 ± 0.56	0.48
Furanofukinin	0.89 ± 0.80	0.5
Glandulone B	0.88 ± 1.27	0.49
Methyl (9Z)-6'-oxo-6,5'-diapo-6-carotenoate	0.86 ± 1.28	ns
4,5-Dihydrovomifoliol	0.84 ± 0.58	0.39
Methyl geranate	0.83 ± 0.65	0.61
Ganoderiol C	0.82 ± 1.21	ns
Oxysolavetivone	0.81 ± 0.54	0.47

## Data Availability

Data supporting the reported results are available upon request from the corresponding authors.

## Supplementary Materials

The following are available online at https://www.mdpi.com/article/10.3390/antiox10101580/s1: Table S1: Statistical differences in antioxidant capacity and carotenoids content among the 20 pumpkin samples; Table S2: Dietary terpenoids annotated by using UHPLC-Orbitrap mass spectrometry in all pumpkin samples; Table S3: Pumpkins’ weight, length, and diameter; Figure S1: Images of Serbian pumpkins with scale bars; Figure S2: Images of non-Serbian pumpkins with scale bars.
