# Supplementary material for: Comparative In Vitro Antioxidant Capacity and Terpenoid Profiling of Pumpkin Fruit Pulps from a Serbian Cucurbita maxima and Cucurbita moschata Breeding Collection
Source: Antioxidants (Basel). 2021 Oct 7;10(10):1580. doi: 10.3390/antiox10101580 (PMC8533216; doi:10.3390/antiox10101580)
Supplement: Supplementary file 1 [file antioxidants-10-01580-s001.zip › Table S1 .pdf]

| | ABTS | FRAP | Carotenoid content (mg/g) |
|---|---|---|---|
| | Trolox equivalent (mM) | Ascorbic acid equivalent (mM) | |
| 1 | 0.11 ± 0.005fg | 0.04 ± 0.006jk | 0.27 ± 0.06hij |
| 2 | 0.20 ± 0.010a | 0.07 ± 0.010bcd | 0.34 ± 0.02gh |
| 3 | 0.15 ± 0.002c | 0.06 ± 0.003cdefg | 0.67 ± 0.14f |
| 4 | 0.17 ± 0.003b | 0.08 ± 0.003bc | 0.84 ± 0.08e |
| 13 | 0.10 ± 0.004fgh | 0.04 ± 0.009ijk | 0.18 ± 0.02ijk |
| 20 | 0.14 ± 0.004cd | 0.07 ± 0.006bcdef | 0.29 ± 0.05hi |
| 22 | 0.13 ± 0.006de | 0.06 ± 0.003cdefg | 2.09 ± 0.11a |
| 23 | 0.11 ± 0.003f | 0.07 ± 0.003bcdef | 0.46 ± 0.09g |
| 26 | 0.09 ± 0.006h | 0.05 ± 0.009ghijk | 0.34 ± 0.04gh |
| 67 | 0.11 ± 0.003f | 0.08 ± 0.004ab | 0.15 ± 0.02jk |
| 173 | 0.13 ± 0.005e | 0.07 ± 0.003bcde | 1.17 ± 0.05c |
| 177 | 0.05 ± 0.003i | 0.04 ± 0.008k | 1.45 ± 0.06b |
| 212 | 0.16 ± 0.004b | 0.10 ± 0.010a | 1.01 ± 0.10d |
| 245 | 0.05 ± 0.002i | 0.05 ± 0.007ghijk | 0.17 ± 0.00c |
| 369 | 0.10 ± 0.005fg | 0.06 ± 0.008efghij | 0.36 ± 0.02gh |
| 370 | 0.17 ± 0.004b | 0.06 ± 0.001defg | 0.28 ± 0.06hij |
| 373 | 0.11 ± 0.008fg | 0.06 ± 0.006defghi | 0.24 ± 0.08hij |
| 377 | 0.12 ± 0.004f | 0.05 ± 0.009hijk | 0.09 ± 0.01k |
| 381 | 0.09 ± 0.004gh | 0.05 ± 0.004hijk | 0.28 ± 0.07hij |
| 384 | 0.14 ± 0.002cd | 0.06 ± 0.003fghij | 0.04 ± 0.01k |

The results were statistically analyzed using EXCEL with installed DSAASTAT add-in. In order to determine statistically significant differences between varieties, an analysis of variance was made. Multiple comparisons analyses were performed using Tukey HSD method ($p < 0.05$).
