# Supplementary material for: Comparative In Vitro Antioxidant Capacity and Terpenoid Profiling of Pumpkin Fruit Pulps from a Serbian Cucurbita maxima and Cucurbita moschata Breeding Collection
Source: Antioxidants (Basel). 2021 Oct 7;10(10):1580. doi: 10.3390/antiox10101580 (PMC8533216; doi:10.3390/antiox10101580)
Supplement: Supplementary file 1 [file antioxidants-10-01580-s001.zip › Table S3.pdf]

Pumpkins Weight, Length and Diameter

Plot number	Accession	Fruit weight (kg)	Fruit length (cm)	Fruit diameter (cm)
1	Max 113	4.95	28.5	27.82
2	Max 118-1	3.78	18.77	22.02
3	Max 118-2	8.02	29.76	33.88
4	Max 117	5.97	24.5	29.81
13	Max 37	4.64	23.2	19.78
20	Mo 31	1.88	33.01	13.05
22	Mo 39	1.85	30.5	13.11
23	Mo 2	2.12	30.04	16.56
26	Mo 29	2.37	27.12	12.35
67	Hokk 54-1	1.02	11.02	17.25
173	Max 35	3.65	12.07	29.84
177	Max 65-1	6.33	21.44	27.13
212	Max 1	8.96	38.25	33.14
245	Max 105	13.08	34.58	42.02
369	Mo 29-1	2.73	37.05	16.14
370	Mo 29-2	1.73	31.11	16.6
373	Mo 38	2.06	39.72	13.22
377	Mo 32	2.4	37.15	16.42
381	Mo F1 35 x 38	3.96	30.23	17.06
384	Max 122	6.93	20.17	30.41
