# Supplementary figures and images for: Comparative In Vitro Antioxidant Capacity and Terpenoid Profiling of Pumpkin Fruit Pulps from a Serbian Cucurbita maxima and Cucurbita moschata Breeding Collection
Source: Antioxidants (Basel). 2021 Oct 7;10(10):1580. doi: 10.3390/antiox10101580 (PMC8533216; doi:10.3390/antiox10101580)

2

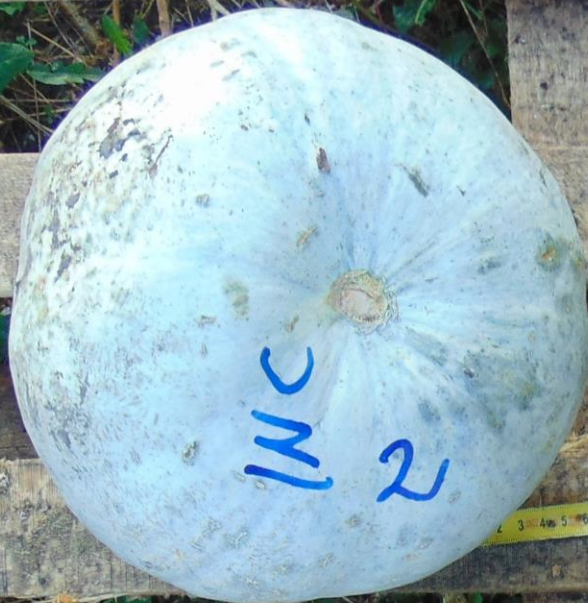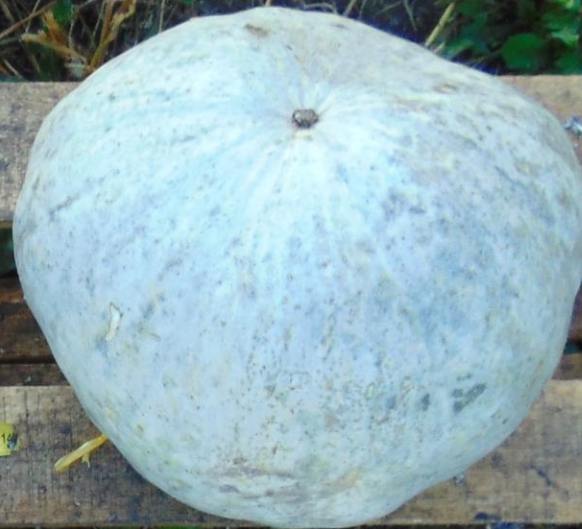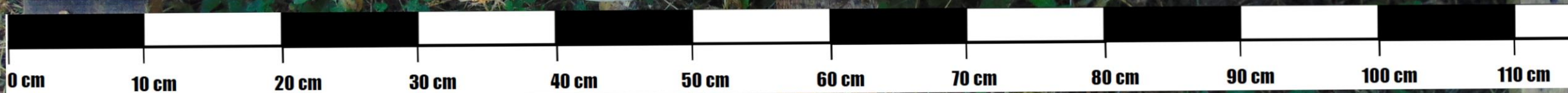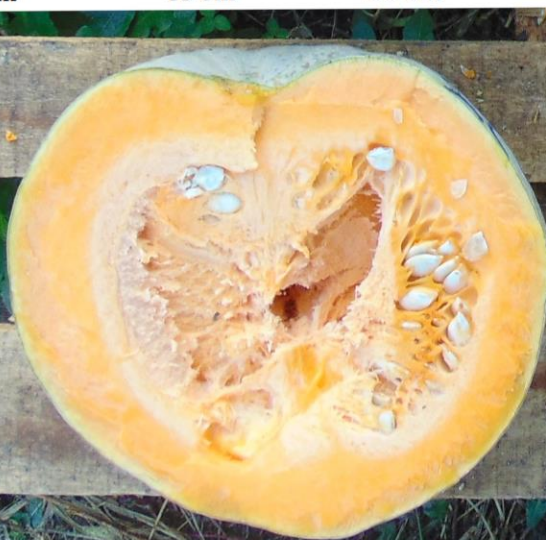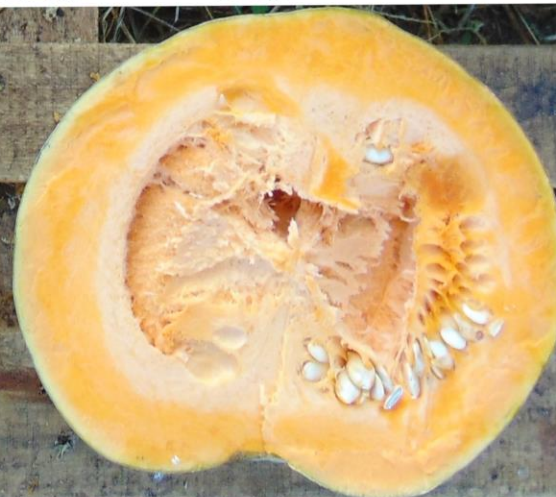

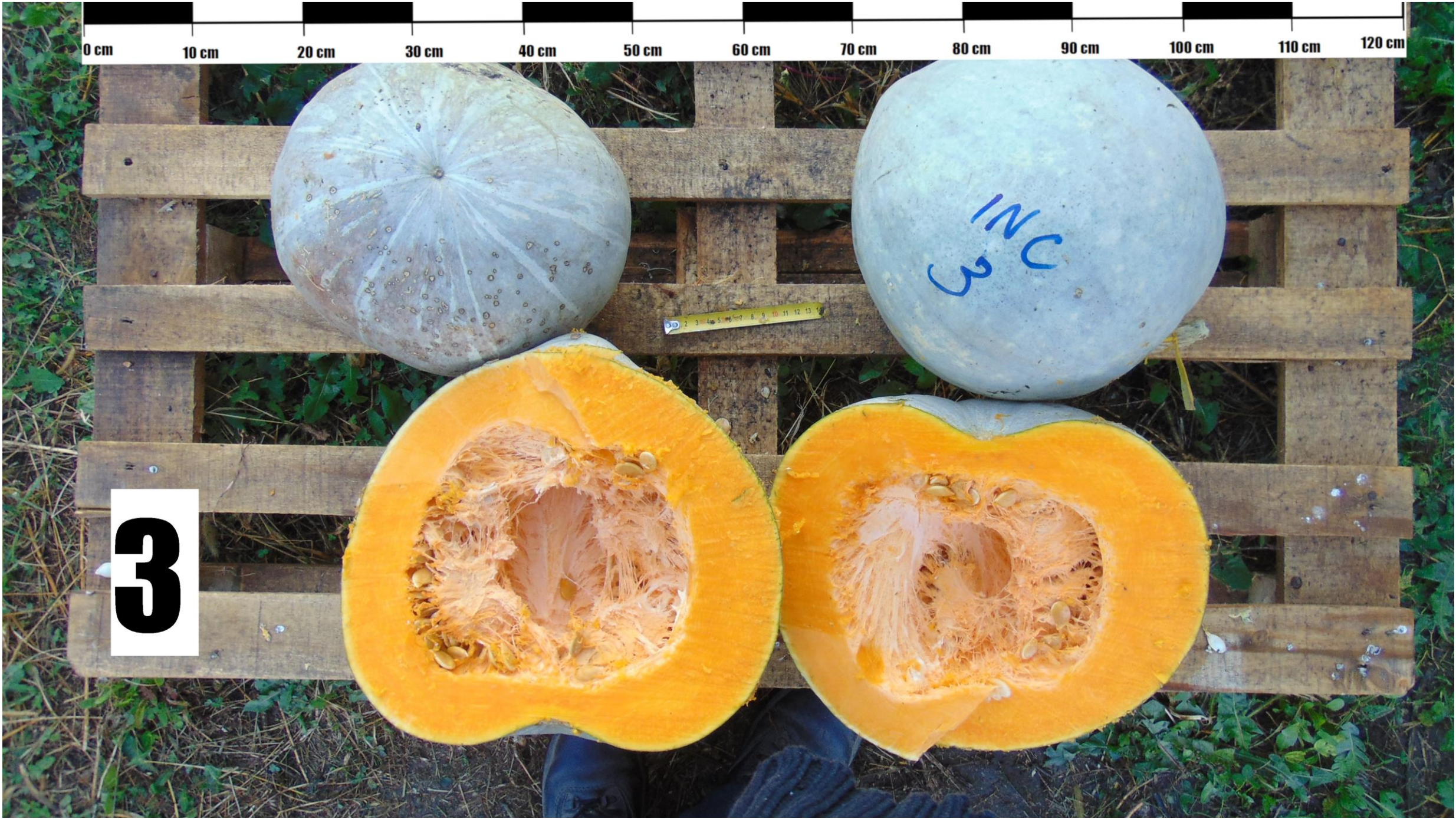

4

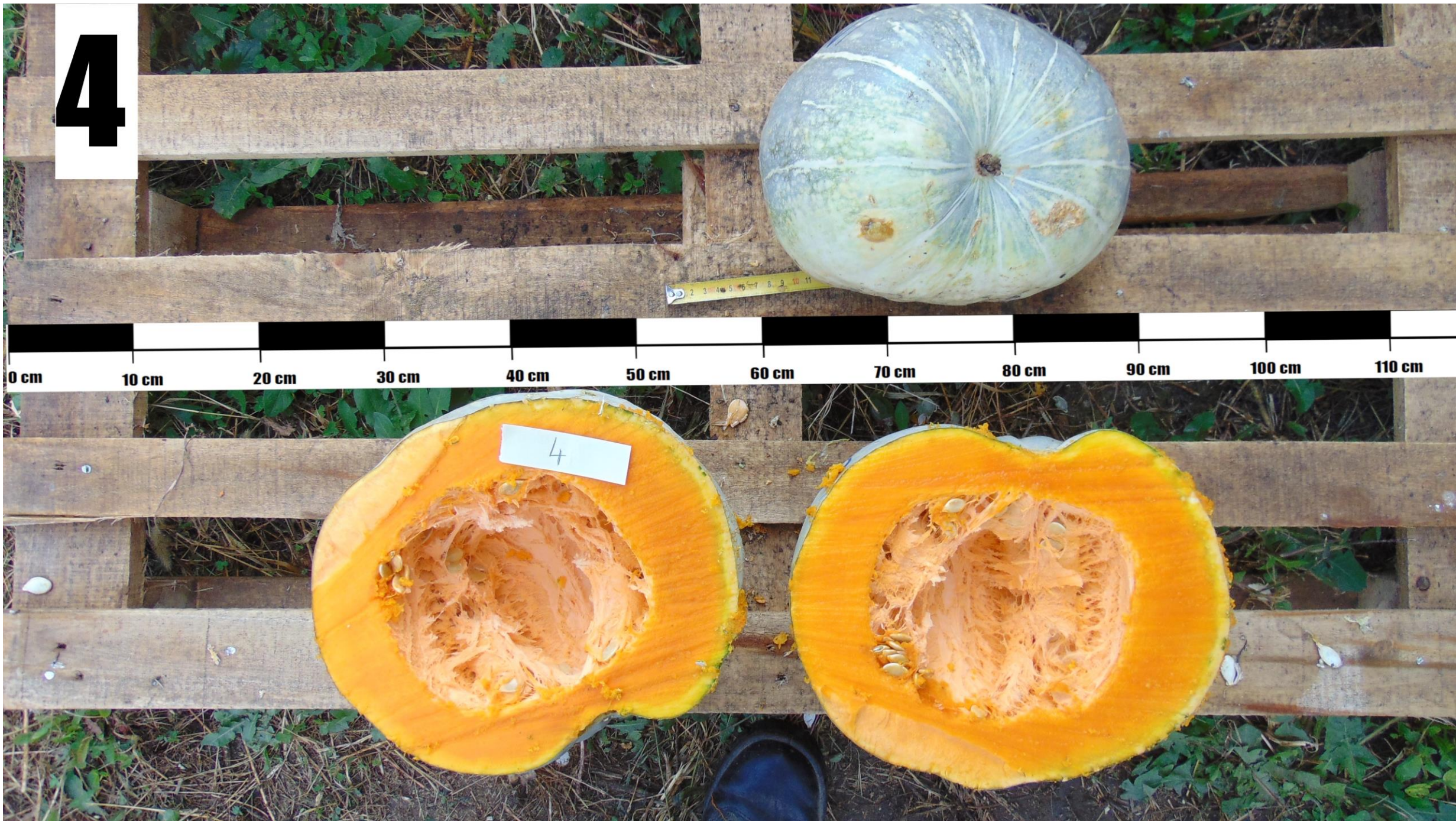

13

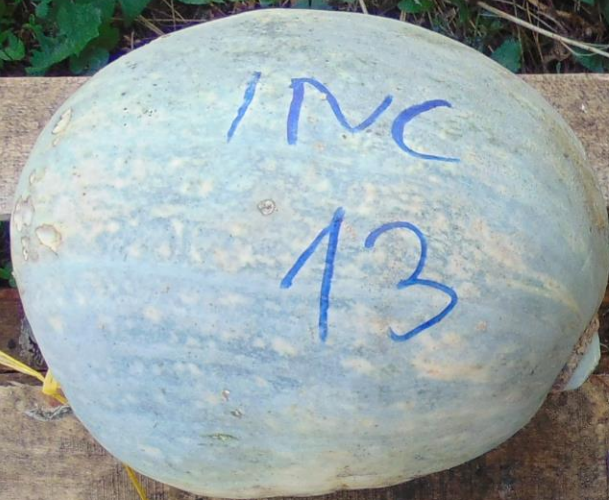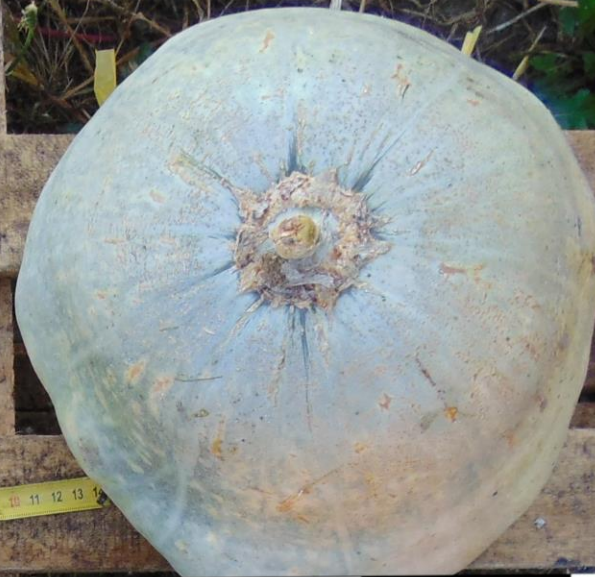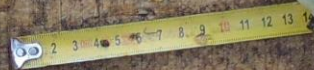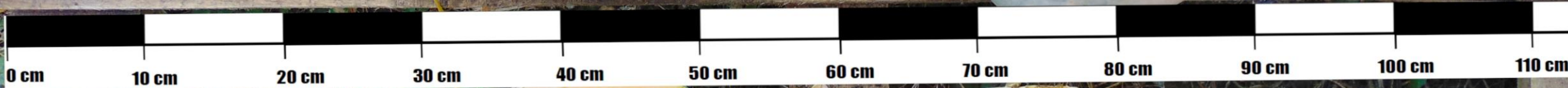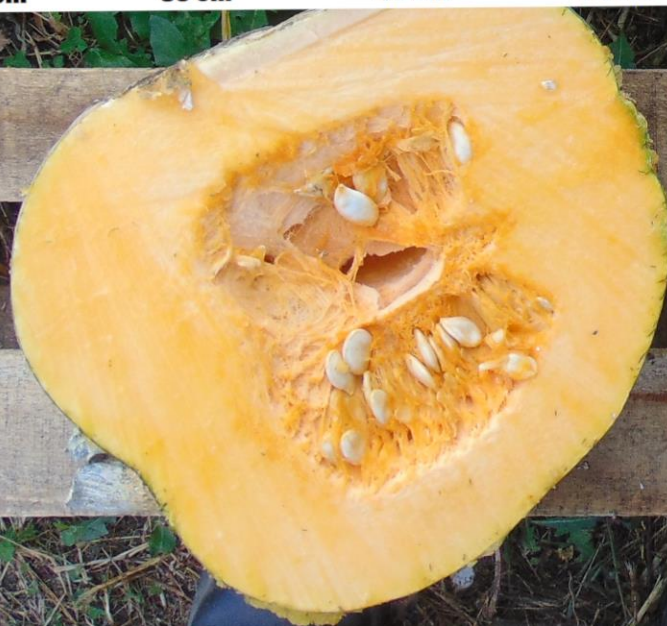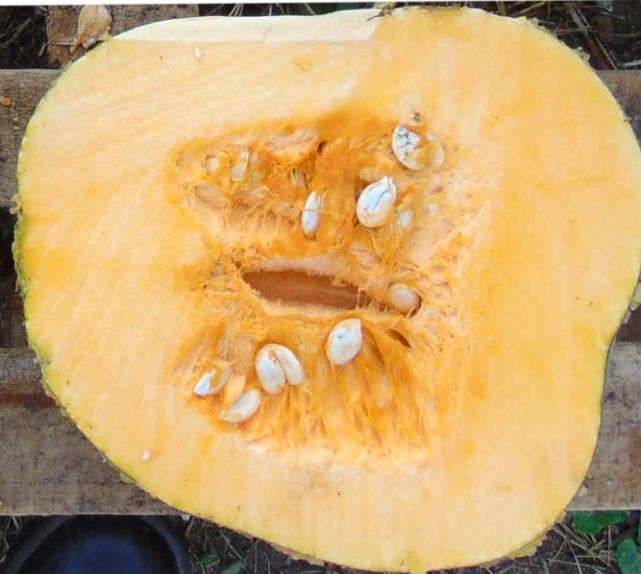

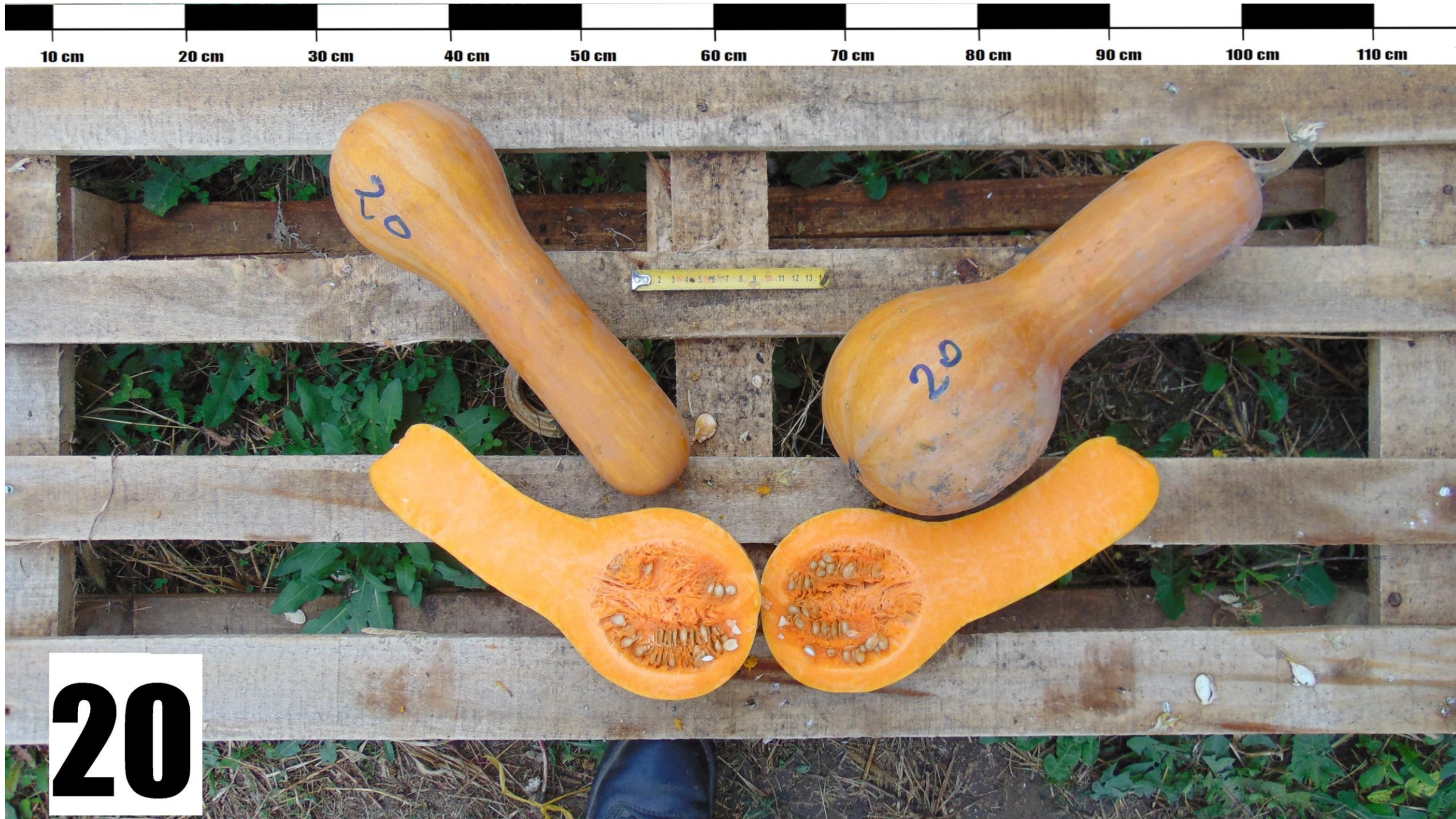

10 cm

20 cm

30 cm

40 cm

50 cm

60 cm

70 cm

80 cm

90 cm

100 cm

110 cm

2 3 4 5 6 7 8 9 10 11 12 13 14

20

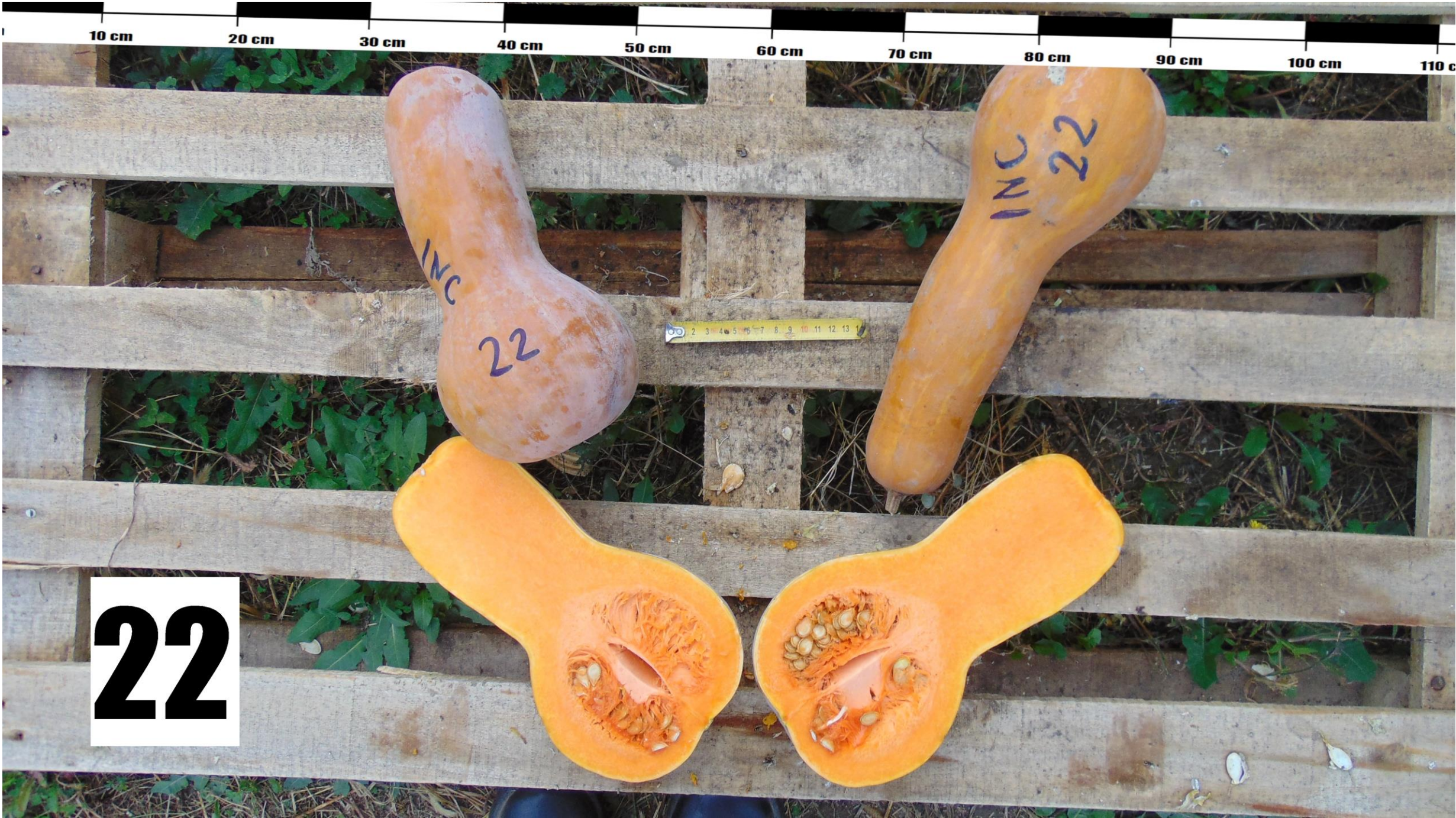

26

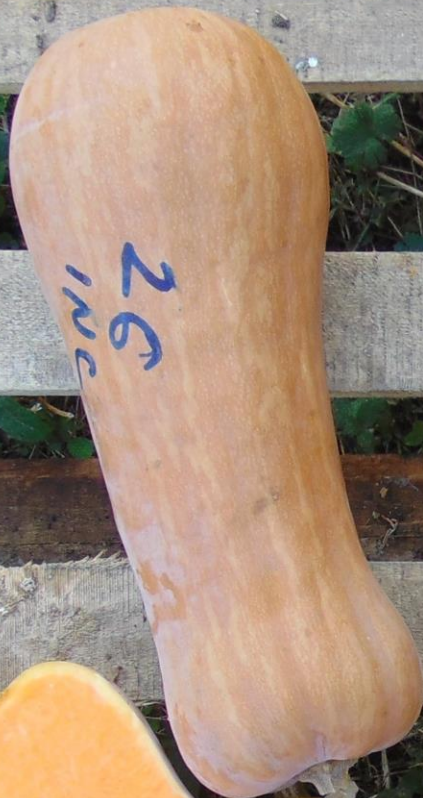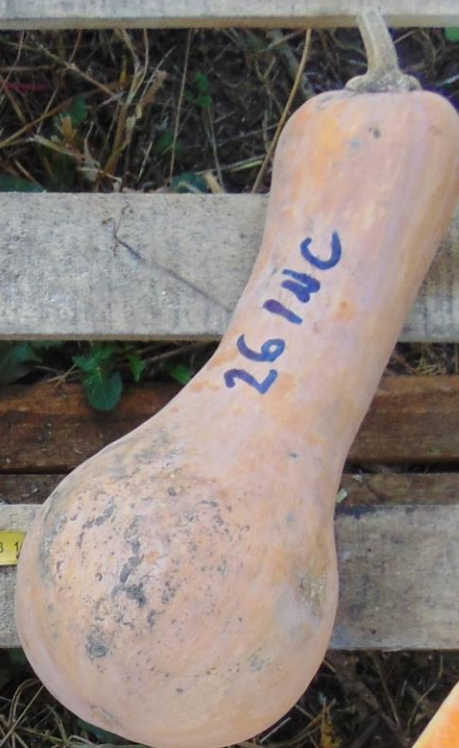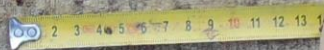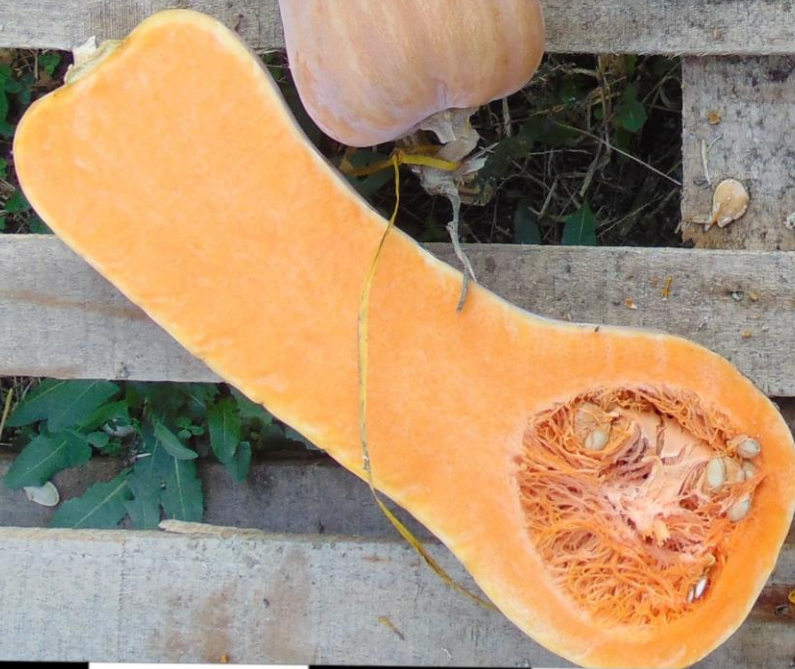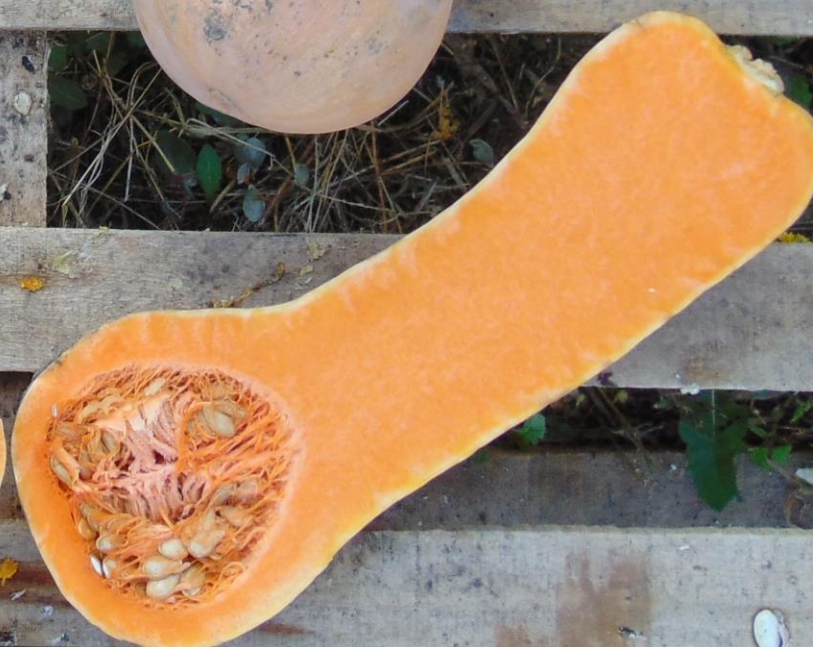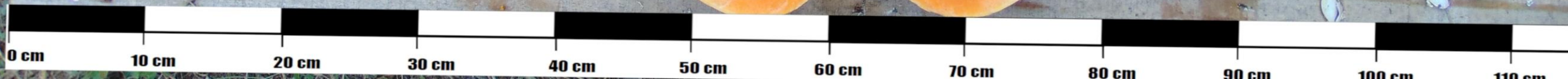

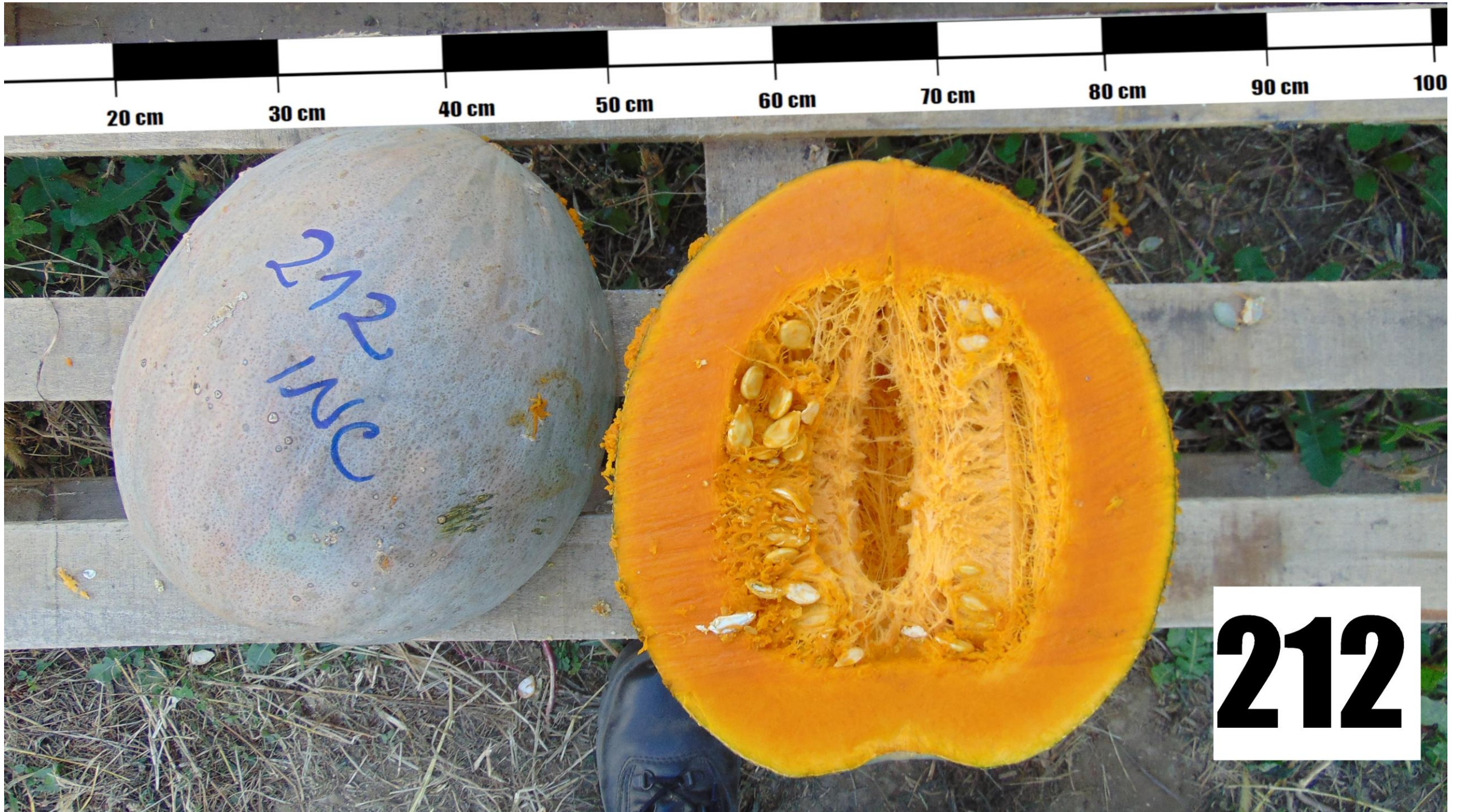

232 MC

212

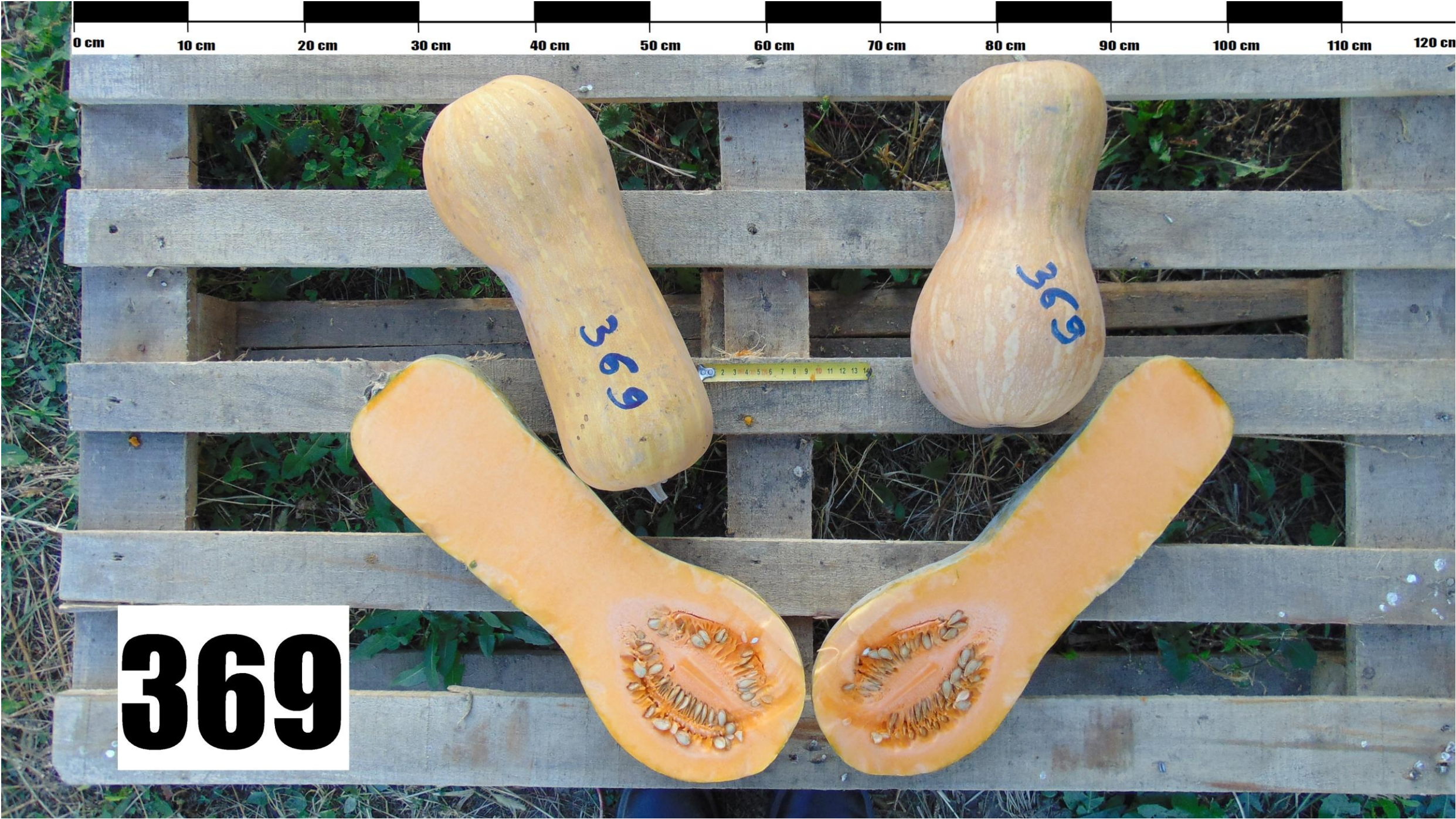

0 cm 10 cm 20 cm 30 cm 40 cm 50 cm 60 cm 70 cm 80 cm 90 cm 100 cm 110 cm 120 cm

369

370

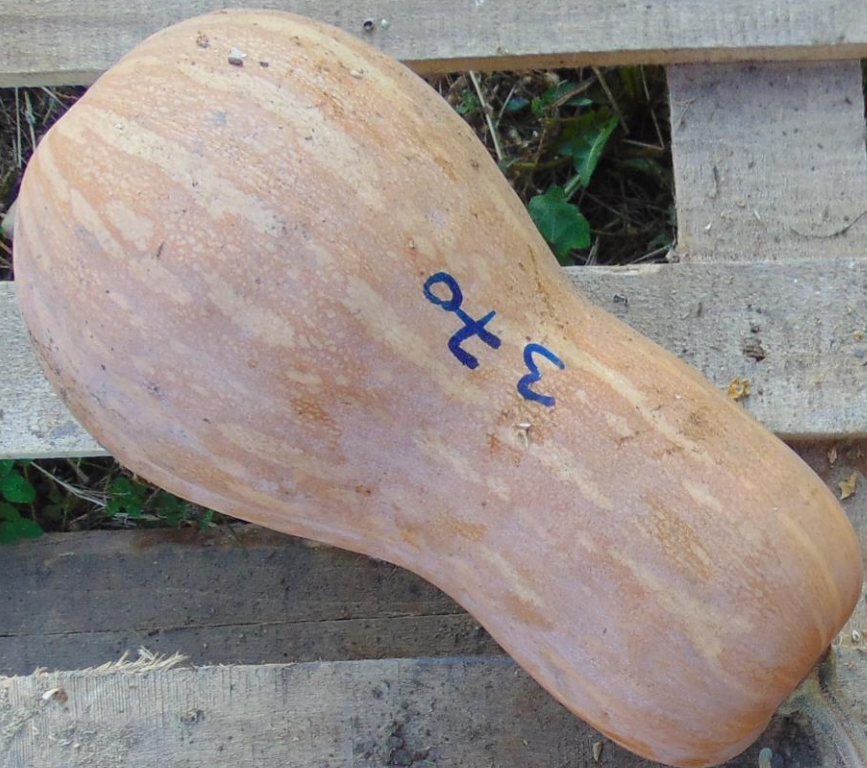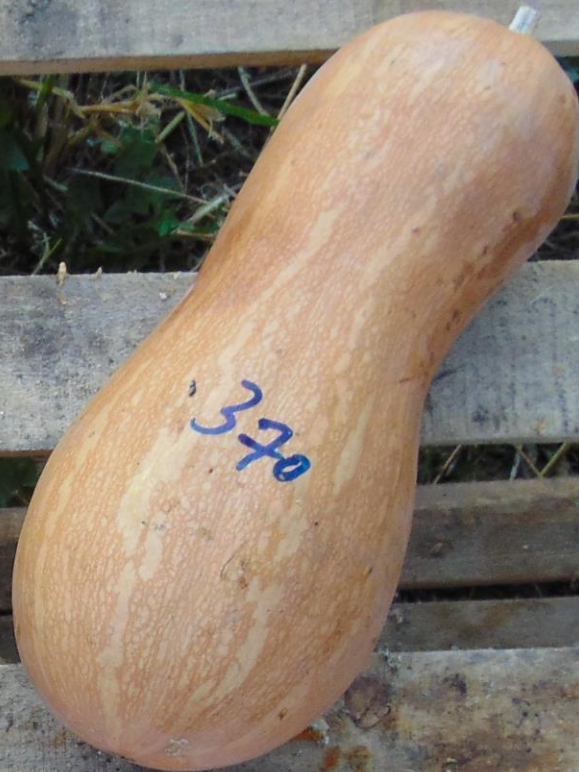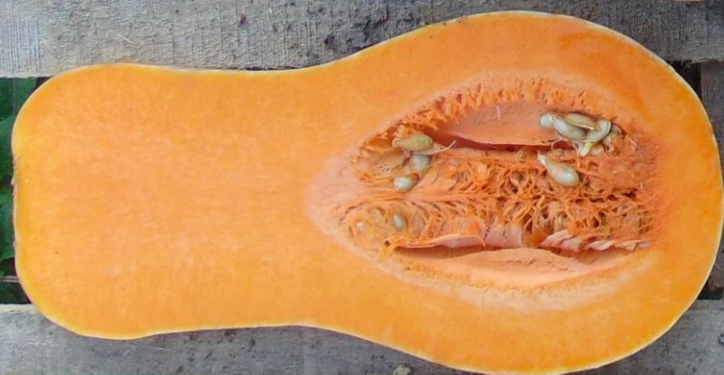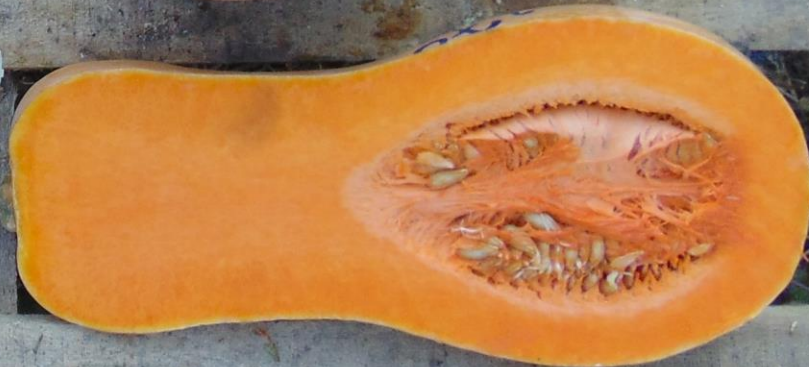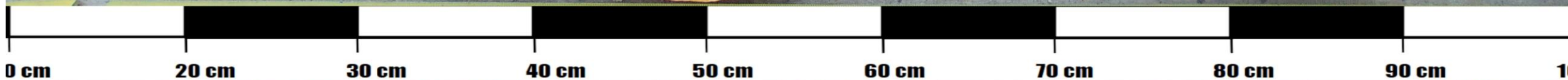

373

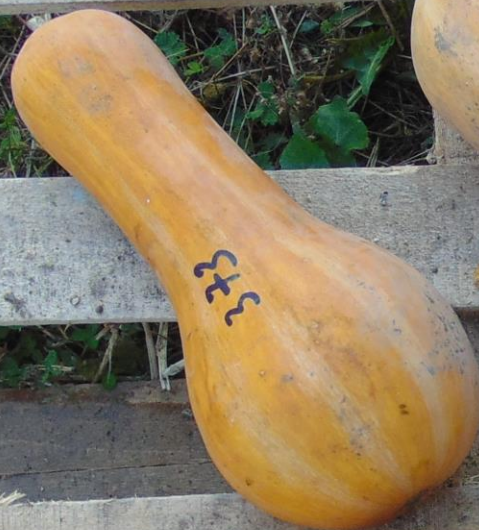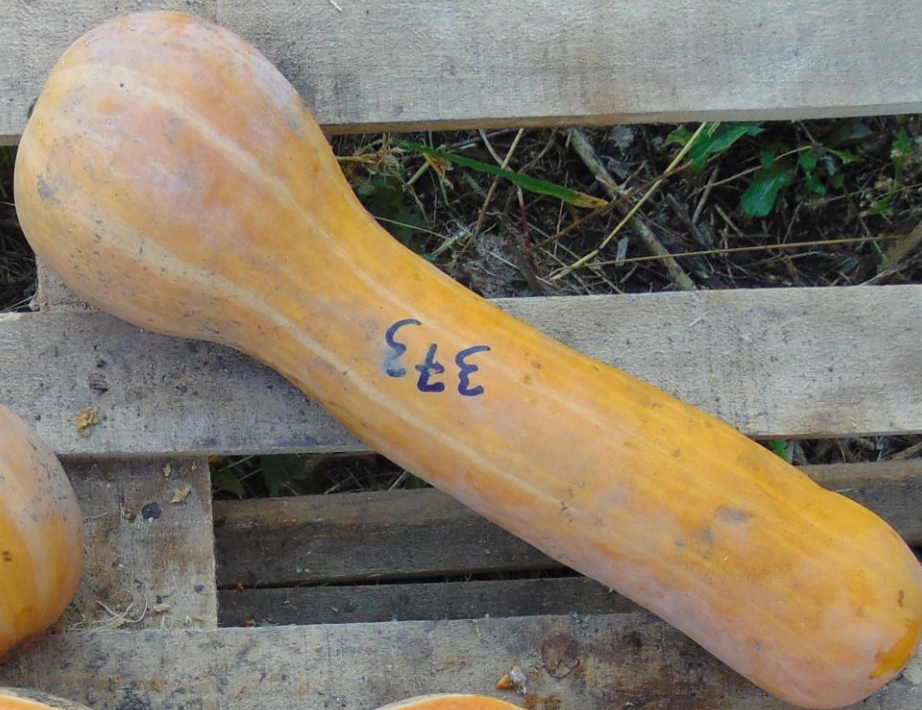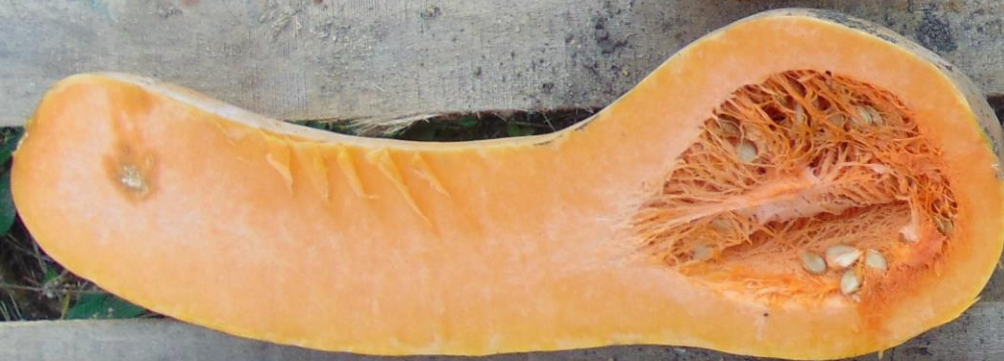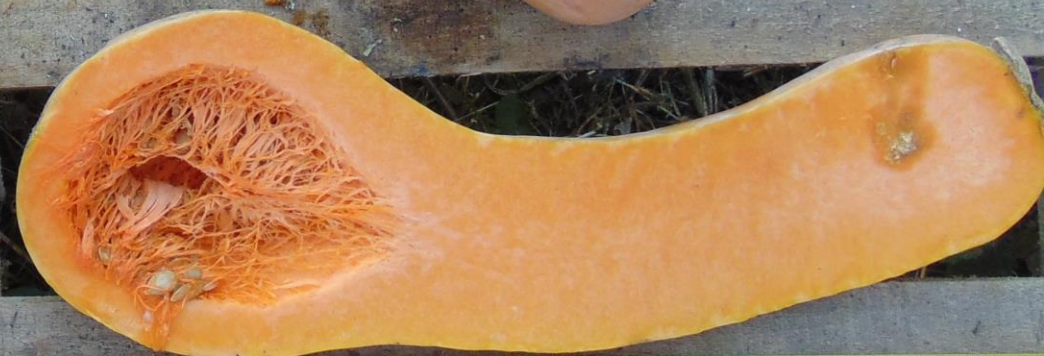

10 cm

20 cm

30 cm

40 cm

50 cm

60 cm

70 cm

80 cm

90 cm

100 cm

110 cm

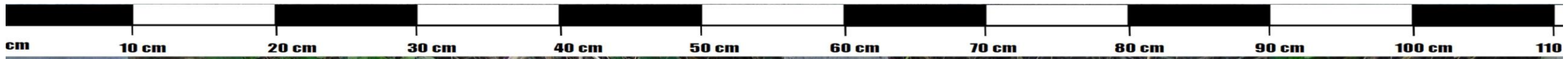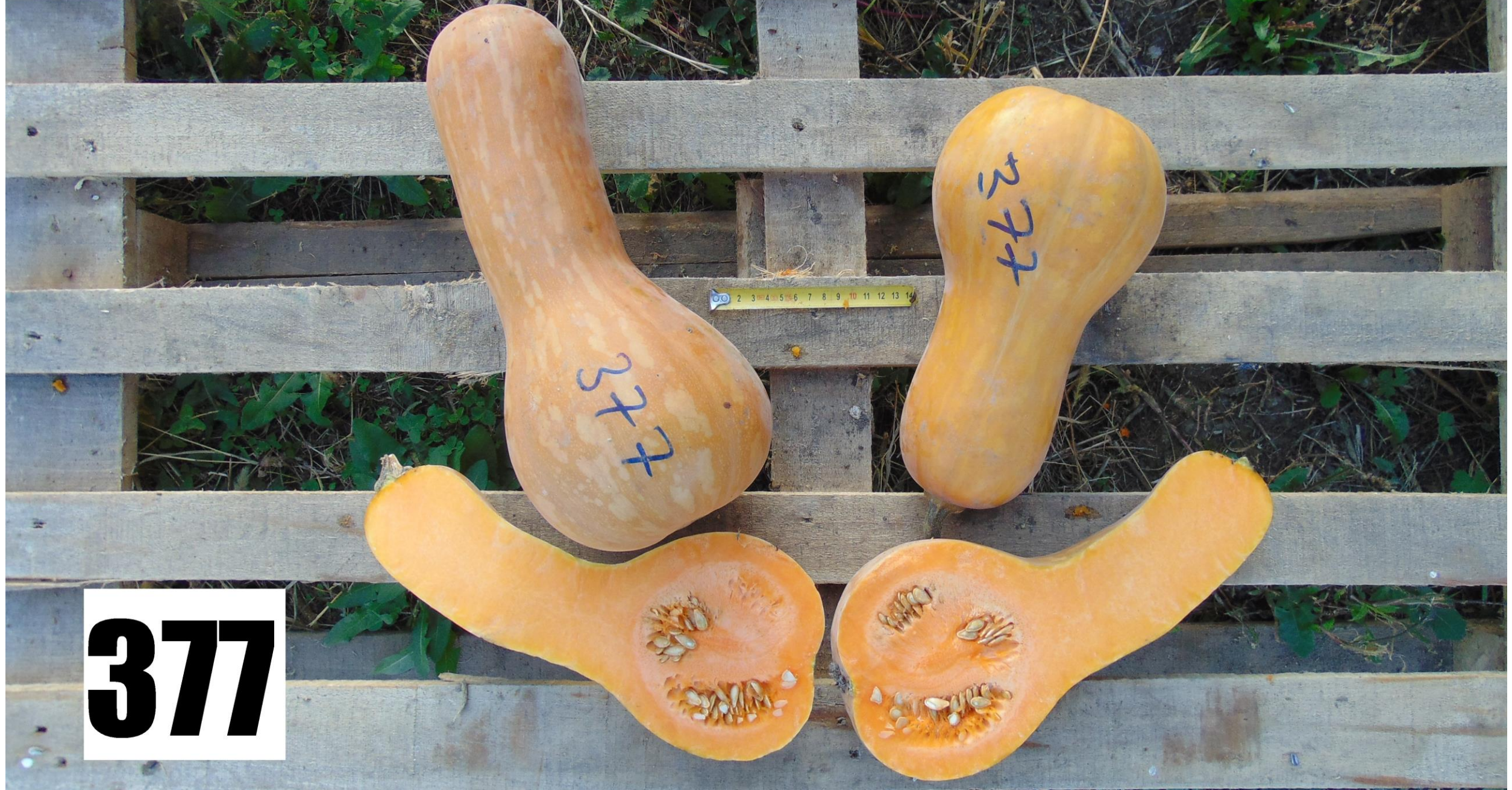

**377**

381

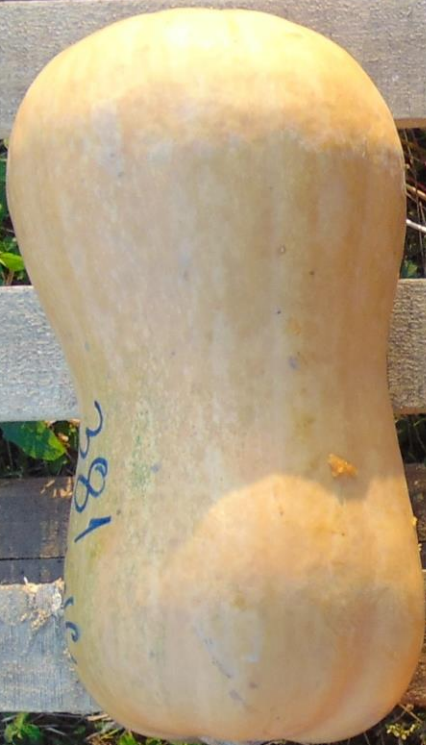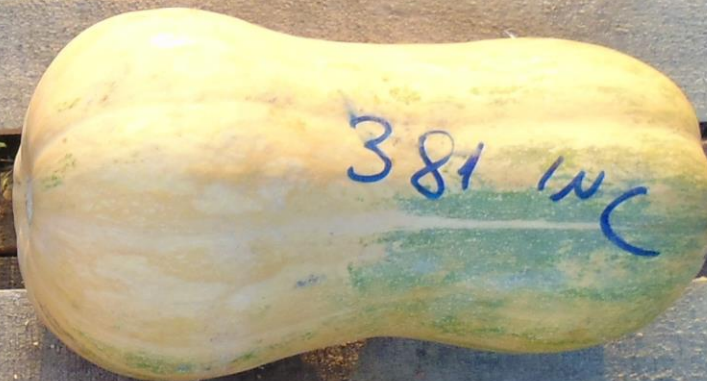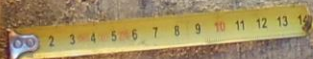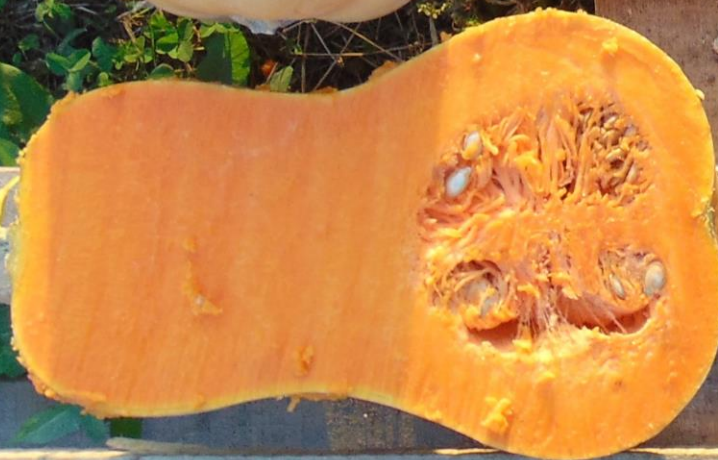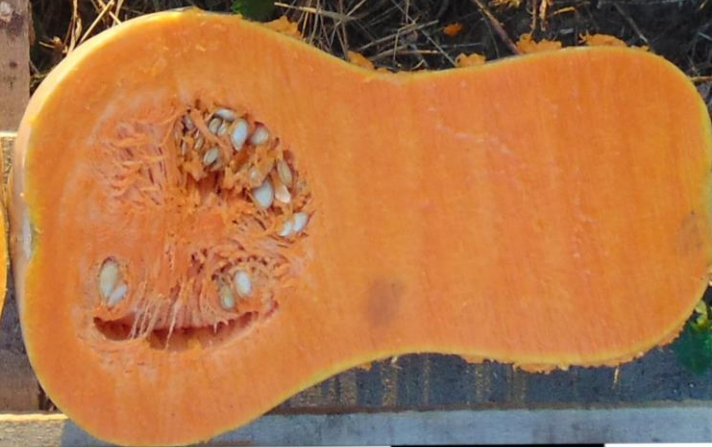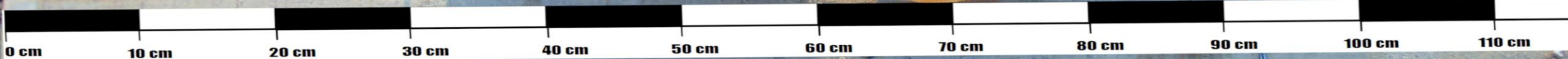

Supplement: Supplementary file 1 [file antioxidants-10-01580-s001.zip › FigS1-Serbian pumpkins.pdf]

1

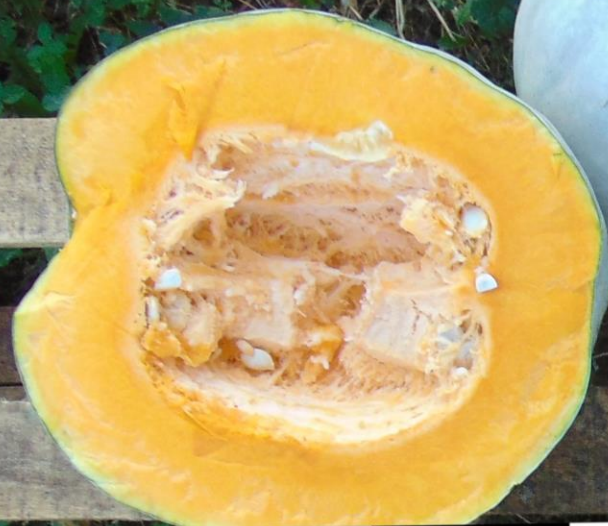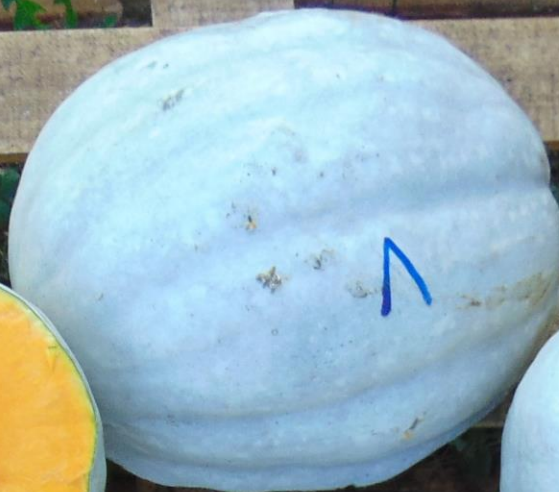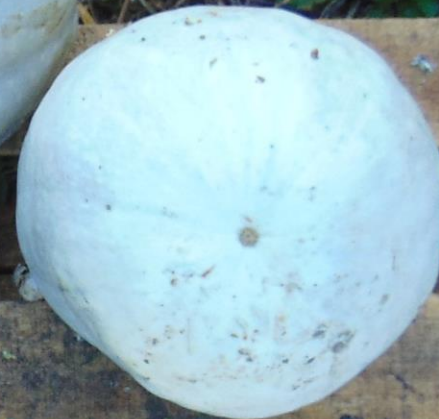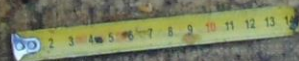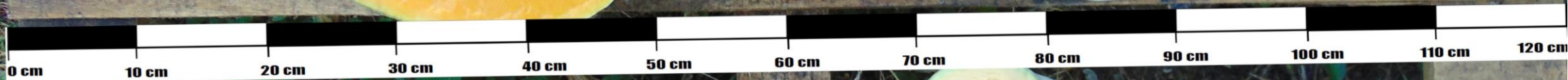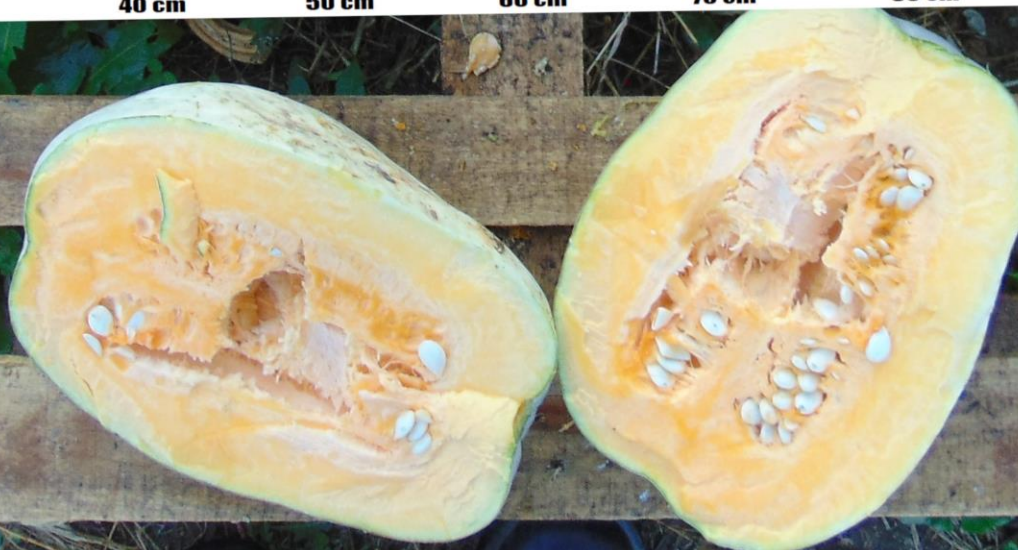

23

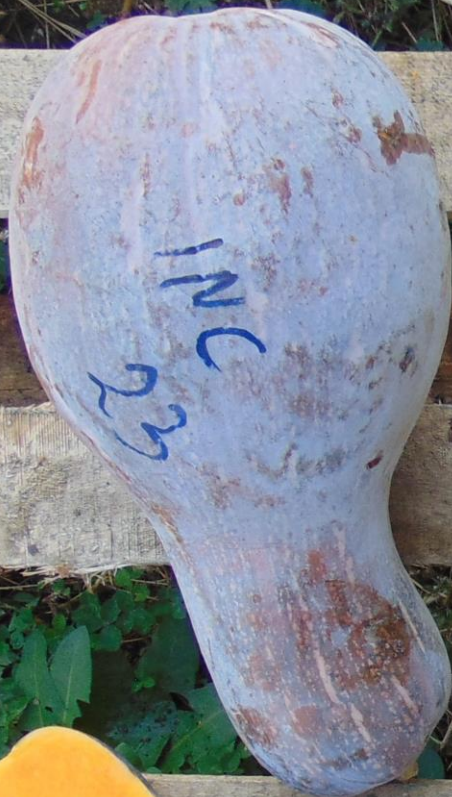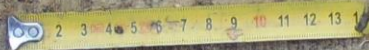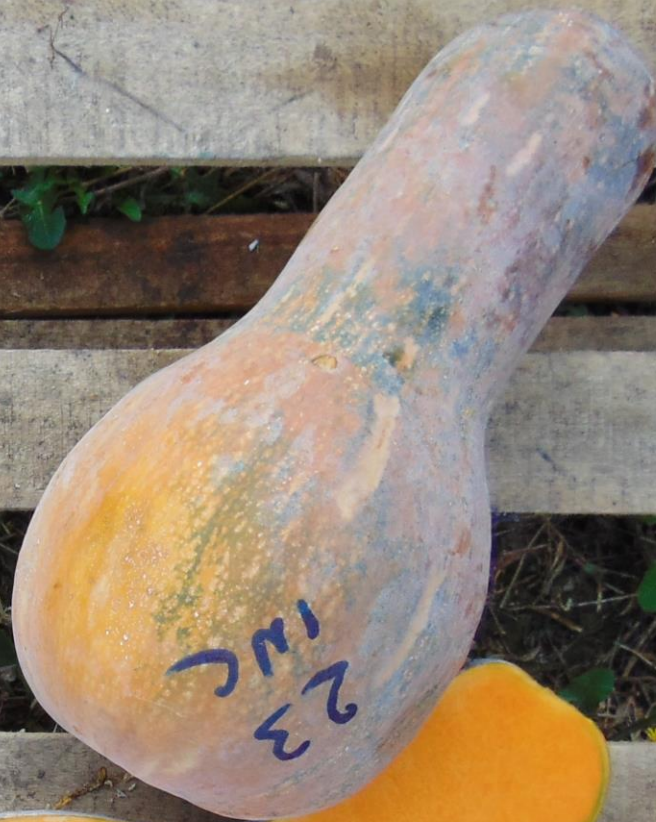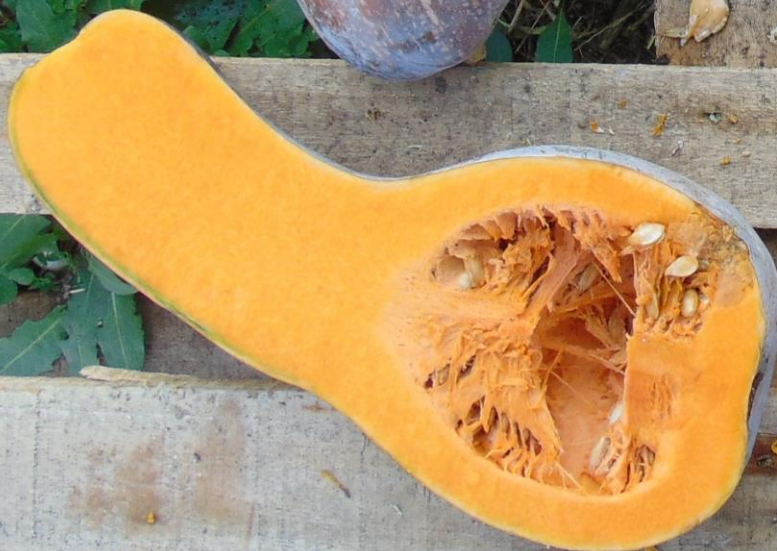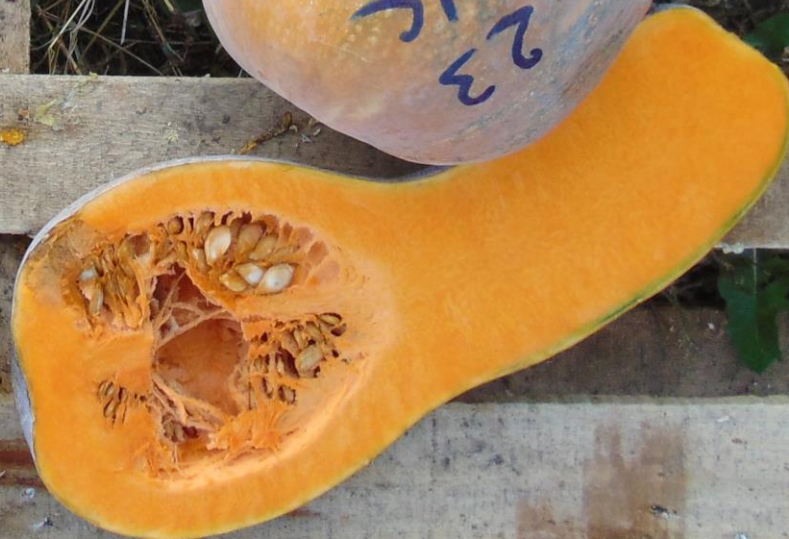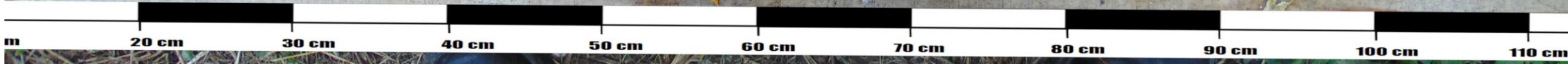

67

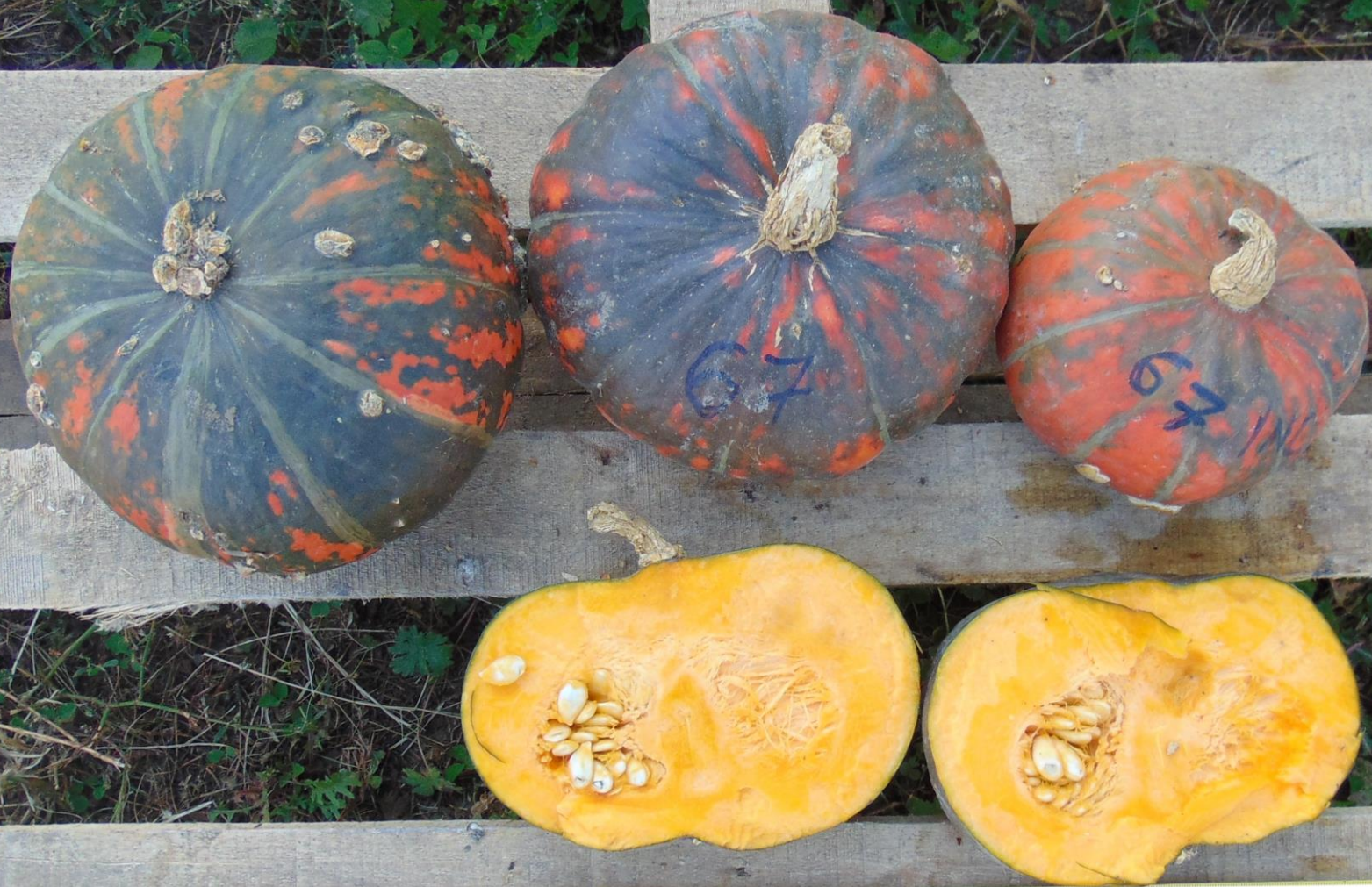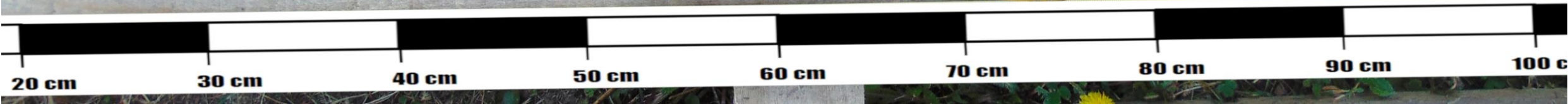

173

173

cm

30 cm

40 cm

50 cm

60 cm

70 cm

80 cm

90 cm

100 cm

177

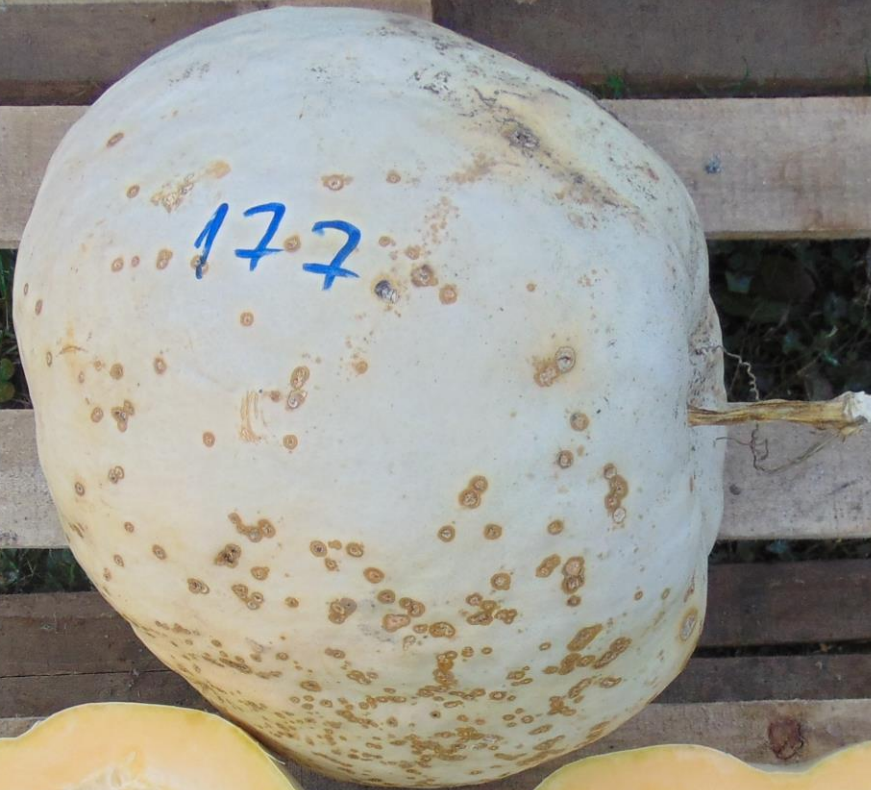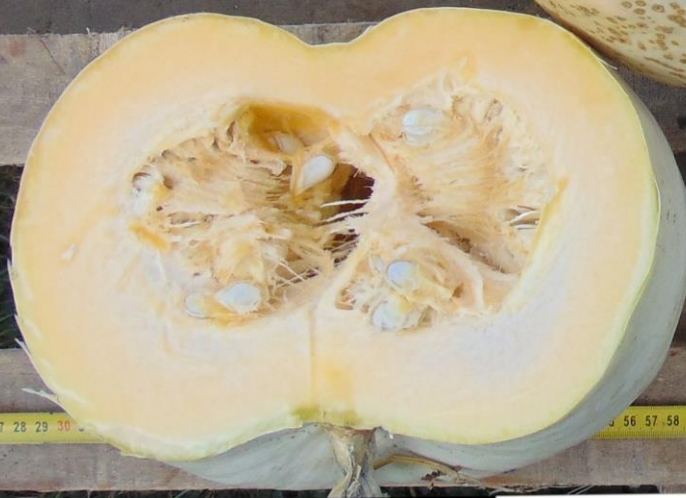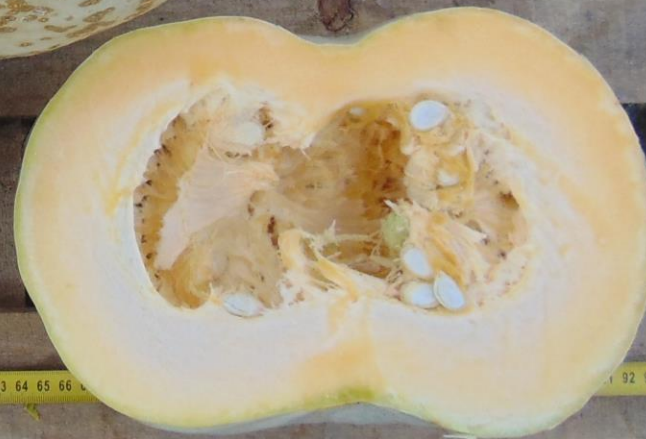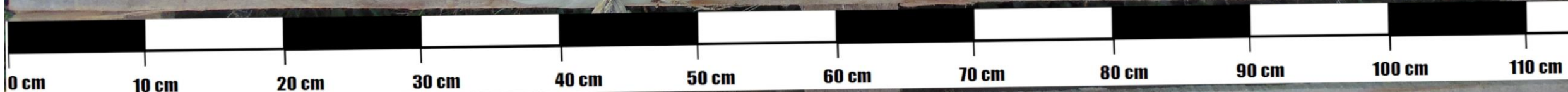

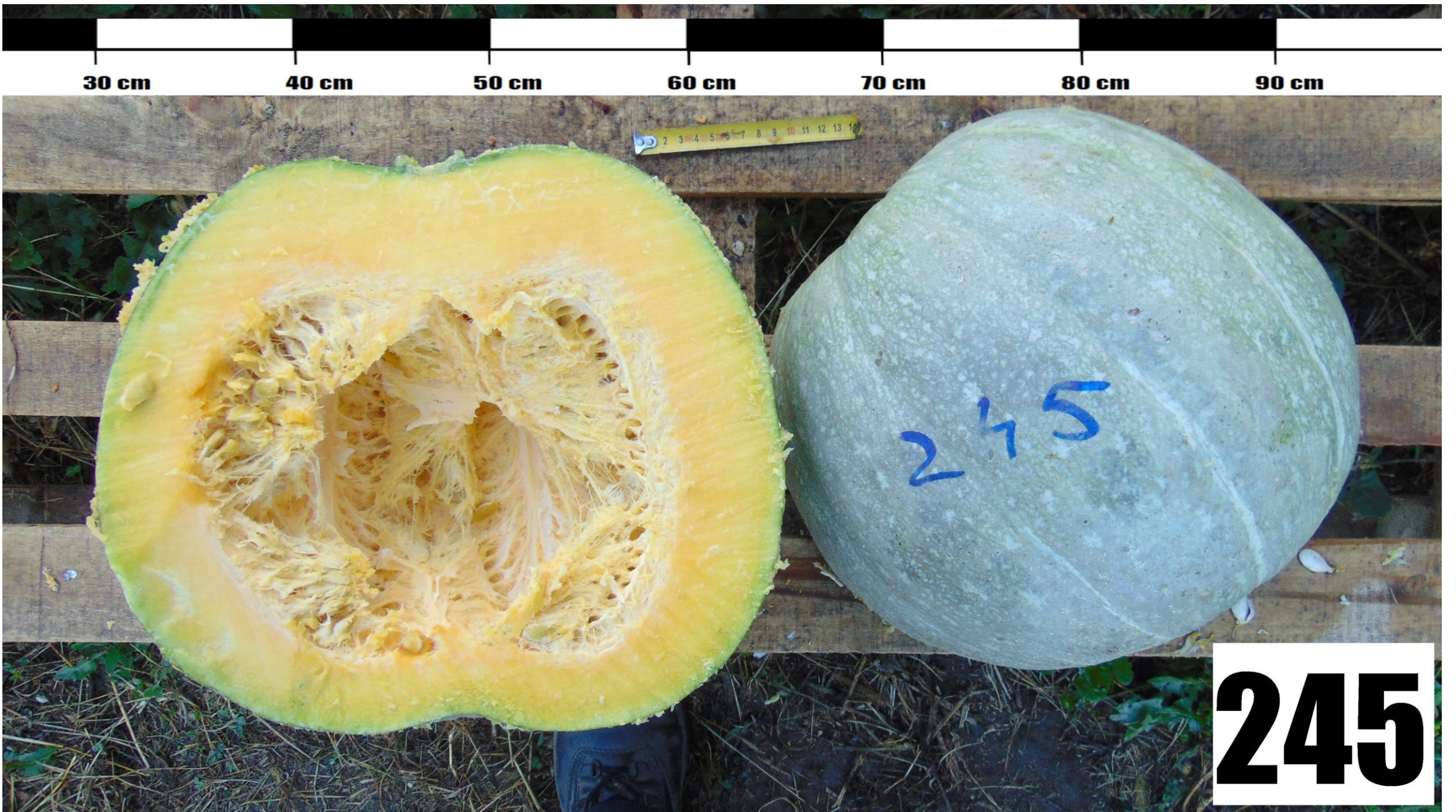

245

245

384

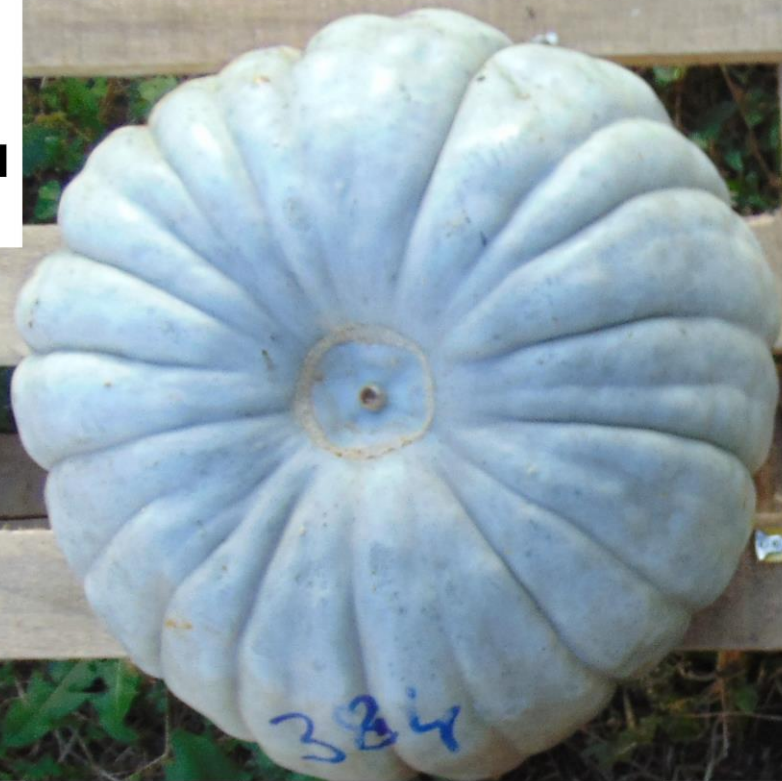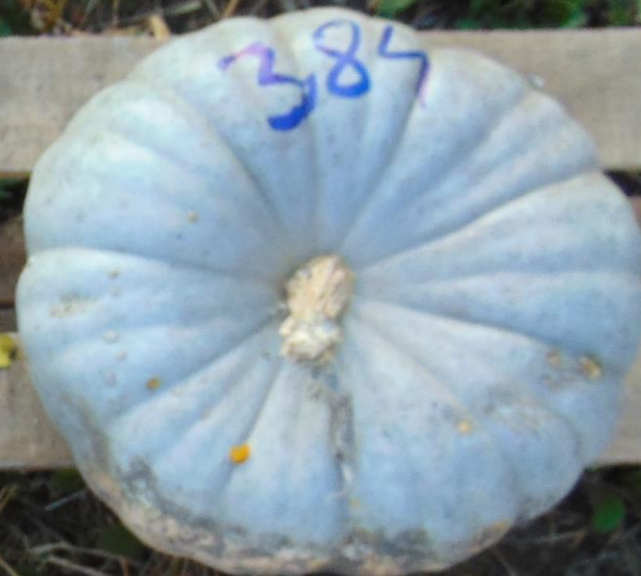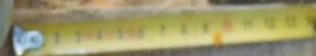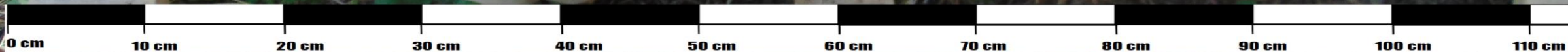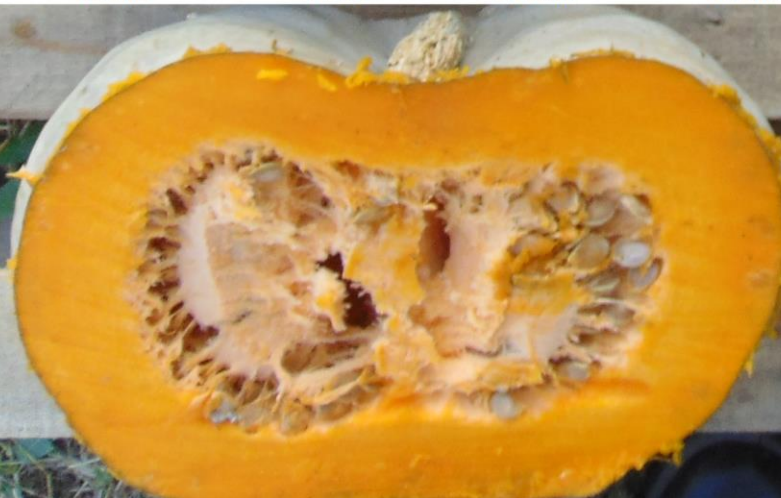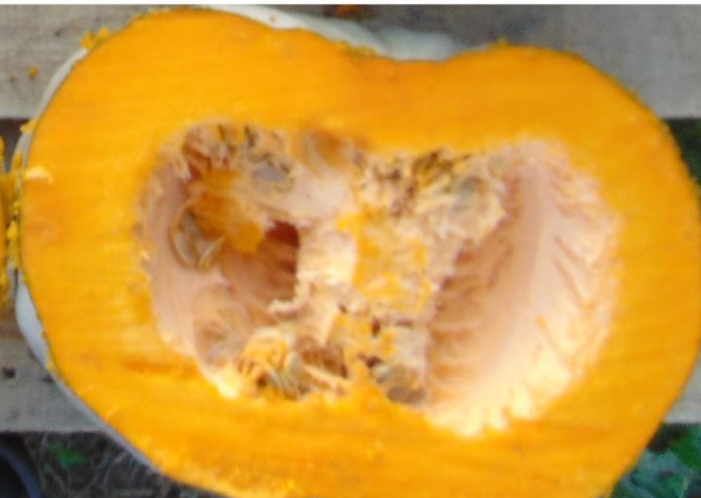

Supplement: Supplementary file 1 [file antioxidants-10-01580-s001.zip › FigS2-non-Serbian pumpkins.pdf]
